# Tripartite degrons confer diversity and specificity on regulated protein degradation in the ubiquitin-proteasome system

**DOI:** 10.1038/ncomms10239

**Published:** 2016-01-06

**Authors:** Mainak Guharoy, Pallab Bhowmick, Mohamed Sallam, Peter Tompa

**Affiliations:** 1 VIB Structural Biology Research Center (SBRC), Vrije Universiteit Brussel (VUB), Building E, Pleinlaan 2, 1050 Brussels, Belgium; 2 Institute of Enzymology, Research Center for Natural Sciences, Hungarian Academy of Sciences, 1117 Budapest, Hungary

## Abstract

Specific signals (degrons) regulate protein turnover mediated by the ubiquitin-proteasome system. Here we systematically analyse known degrons and propose a tripartite model comprising the following: (1) a primary degron (peptide motif) that specifies substrate recognition by cognate E3 ubiquitin ligases, (2) secondary site(s) comprising a single or multiple neighbouring ubiquitinated lysine(s) and (3) a structurally disordered segment that initiates substrate unfolding at the 26S proteasome. Primary degron sequences are conserved among orthologues and occur in structurally disordered regions that undergo E3-induced folding-on-binding. Posttranslational modifications can switch primary degrons into E3-binding-competent states, thereby integrating degradation with signalling pathways. Degradation-linked lysines tend to be located within disordered segments that also initiate substrate degradation by effective proteasomal engagement. Many characterized mutations and alternative isoforms with abrogated degron components are implicated in disease. These effects result from increased protein stability and interactome rewiring. The distributed nature of degrons ensures regulation, specificity and combinatorial control of degradation.

## Abstract (web summary)

Degrons are determinants within proteins that direct programmed degradation by the ubiquitinproteasome system. Here, the authors propose a three-part degron architecture which contains an E3-ligase recognition motif, a ubiquitination site(s), and a disordered site to initiate degradation.

## Graphical abstract

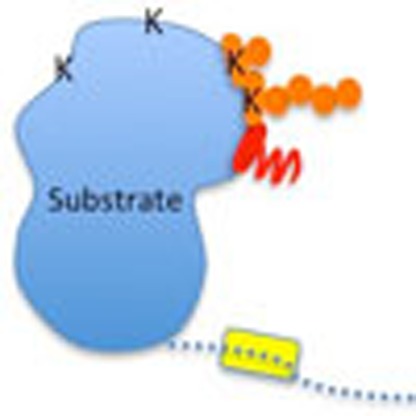

Regulated degradation (Deg) of proteins via the ubiquitin-proteasome system (UPS) is critical for diverse cellular processes such as cell cycle progression, transcription, immune response, signalling, differentiation and growth^1^. Deg is spatio-temporally controlled and needs to be harmonized with protein synthesis and functionality to maintain proteostatic balance and to achieve precise proteome remodelling in response to environmental and intracellular cues. This necessitates an intricate monitoring system capable of recognizing specific signals that mark proteins for Deg (degrons).

A degron has been defined as a protein element that confers metabolic instability^2^. Although seemingly straightforward, its molecular correlates are often difficult to define and the term degron has been used inconsistently in the literature. For example, the degron is often defined as the substrate site that is recognized by E3 ubiquitin ligases and a variety of such degrons (short peptide motifs and specific structural elements) have been characterized^3^. The eukaryotic linear motif resource^4^ also classifies several short, linear motifs (SLiMs) as degrons. In contrast, other studies have indicated the site of polyubiquitination or the polyubiquitin chain itself as the degron^5^. Lys48-ubiquitin linkages form the canonical signal for Deg by the 26S proteasome, although other linkage types may also be recognized^6^. Recently, Matouschek and colleagues^7^ suggested that successful Deg requires another additional element, an intrinsically disordered Deg initiation site on the substrate that facilitates substrate unfolding and entry into the proteasome catalytic core, which is accessible only through a narrow channel^8^. They subsequently suggested that degrons are bipartite, composed of the substrate-bound polyubiquitin tag and an appropriately spaced disordered Deg initiation site^9^.

In this study we focus on signals that activate proteolysis when protein function is no longer required, that is, regulated Deg. To understand substrate commitment and entry into the Deg pathway, here we have systematically analysed known degrons based on multiple data sets and hypothesize that the minimal region necessary and sufficient for UPS-mediated regulated Deg is composed of three substrate elements. The primary degron is a short, linear (peptide) motif located mostly within structurally disordered regions (less often within surface-exposed segments of structured domains) and contains a specific sequence pattern that is recognizable by cognate E3 ligases. The secondary degron is one (or multiple neighbouring) substrate lysine(s) present on a defined surface region of the substrate (ubiquitination zone^10^). These lysines possess certain contextual preferences that favour (poly)ubiquitin conjugation such as a moderate-to-high degree of local structural flexibility and a biased amino acid composition in its neighbourhood. Finally, the tertiary degron is a disordered/locally flexible Deg initiation site located proximal to (or overlapping with) the secondary degron. We demonstrate that known degron components show a significant correlation with intrinsically disordered regions (IDRs) and highly flexible substrate segments. We had previously analysed the manifold regulatory advantages of structural disorder in enzymatic components involved in ubiquitination^11^ and here we suggest that the multi-layered substrate degron architecture reflects the complexity of proteostatic regulation. Thus, this study lays a solid foundation for a model where the blueprint for protein Deg is encoded in a distributed (combinatorial) architecture, determining the diversity and specificity of Deg, and thereby enabling a complex and spatiotemporal rewiring of the interactome.

## Results

### The underappreciated complexity of the degrome

Proteostasis entails a balance between protein synthesis, functional regulation and Deg. The regulatory complexity is clearly apparent at the synthesis and functional levels, but much less data are available detailing the regulatory elements at the level of Deg (Supplementary Fig. 1). The control of protein production at the transcriptional stage involves a complex interplay between transcription factors (TFs) and DNA regulatory elements (for example, promoters, enhancers and silencers). The transcription of ∼20,000 human genes is regulated by ∼1,800 TFs and ∼636,000 genomic binding sites have been mapped for 119 TFs. Overall, the number of DNA regulatory elements in the genome may reach into millions^12^. Following synthesis, a large variety (∼300 types) of posttranslational modifications (PTMs) regulate protein localization, activity and interactions, in a synergistic and combinatorial manner^13^. The numbers of enzymes for certain modifications can be in the hundreds (for example, several hundred human kinases modifying ∼100,000 phosphosites^13^). Further, a plethora of peptide (sequence) motifs are known to direct a diverse range of protein functions (binding, modifications, localization, proteolytic cleavage and so on) and their total number has been estimated to be around one million in the human proteome^13^.

E3 ubiquitin ligases confer specificity in selecting substrates for UPS-mediated Deg, and consistent with a balance between the regulatory complexity of protein synthesis and functions *vis-à-vis* Deg the estimated number of human E3s is ∼600 (ref. 11). This is commensurable with the numbers of TFs and modification enzymes, for example, kinases. However, the balance breaks down when we observe the paucity of characterized E3-specific primary degrons. The number of SLiM-type degrons identified thus far is only 28, of which 25 types are found in human proteins (Table 1 and Supplementary Table 1). Experimental validation is available only for a limited number of corresponding substrates (93 human substrates; Supplementary Table 2) and even with predicted substrates (based on permissive criteria) no >30% of the human proteome can be putatively covered (Supplementary Fig. 1). Further, some of these motifs are highly degenerate (for example, the DBOX and SPOP sequence patterns); thus, even after filtering the number of false predictions is likely to be high and significant exploratory research will be required to validate these predicted candidate proteins as true substrates.

**Figure 1:**
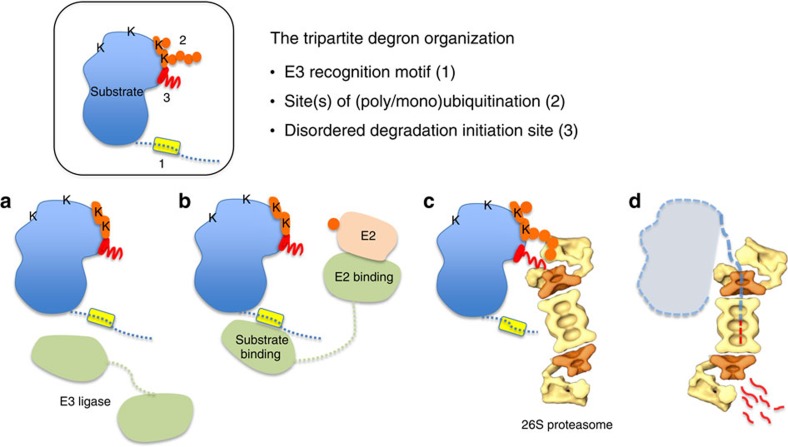
The tripartite degron architecture. Schematic of a substrate protein (box) with the degron components as indicated: primary degron (yellow), lysine(s) and ubiquitin chain(s) (orange), and the disordered Deg initiation site (red). Lysines (K) are indicated. (**a**) Specific recognition of substrate by its cognate E3 ligase (pale green) mediated by the primary degron. (**b**) The E3–E2 (light pink) complex catalyses formation of poly/mono-Ub chain(s) (orange circles) on appropriate acceptor lysine(s). (**c**) The ubiquitinated substrate is recognized by the regulatory subunit(s) of the 26S proteasome. This involves simultaneous recognition of ubiquitin chain(s) and binding to a disordered Deg initiation site that is required for (**d**) local unfolding and transfer into the proteolytic core of the proteasome. The model of the proteasome has been adapted from ref. 67.

In principle, one might expect many more E3 ligases to function by the recognition of a specific SLiM as a primary degron; therefore, the number of unique degrons should be much larger than the current number (that is, 28). Thus, a large part of the ‘degrome' (that is, the full complement of degrons) remains to be explored. We propose that (in addition to as-yet uncharacterized primary degron types and uncharacterized substrates carrying known primary degrons), the regulatory complexity in Deg arises from a tripartite (distributed) degron architecture (Fig. 1), which enables a combinatorial use of degron components making the degrome commensurable in complexity with transcriptional and posttranslational regulatory elements in the proteome.

However, it should be clarified that although many more peptide (SLiM type) primary degrons may be anticipated, not all E3 ligases necessarily bind to peptide degrons. For example, the E3 ligase listerin/Ltn1 forms part of the large ribosomal subunit-associated quality control complex that facilitates translational surveillance in eukaryotes by ubiquitin-tagging defective polypeptides from stalled ribosomes^14^. In another example, a distributed structural degron dispersed across 523 residues of the amino-terminal transmembrane domain of yeast 3-hydroxy-3-methylglutaryl-coenzyme A reductase isozyme Hmg2p is required for its Hrd1-dependent regulated Deg^15^. It is not clear at the moment what fraction of E3 ligases use peptide degrons for substrate recognition.

### Primary degrons specify substrate recognition by E3 ligases

**Figure 2:**
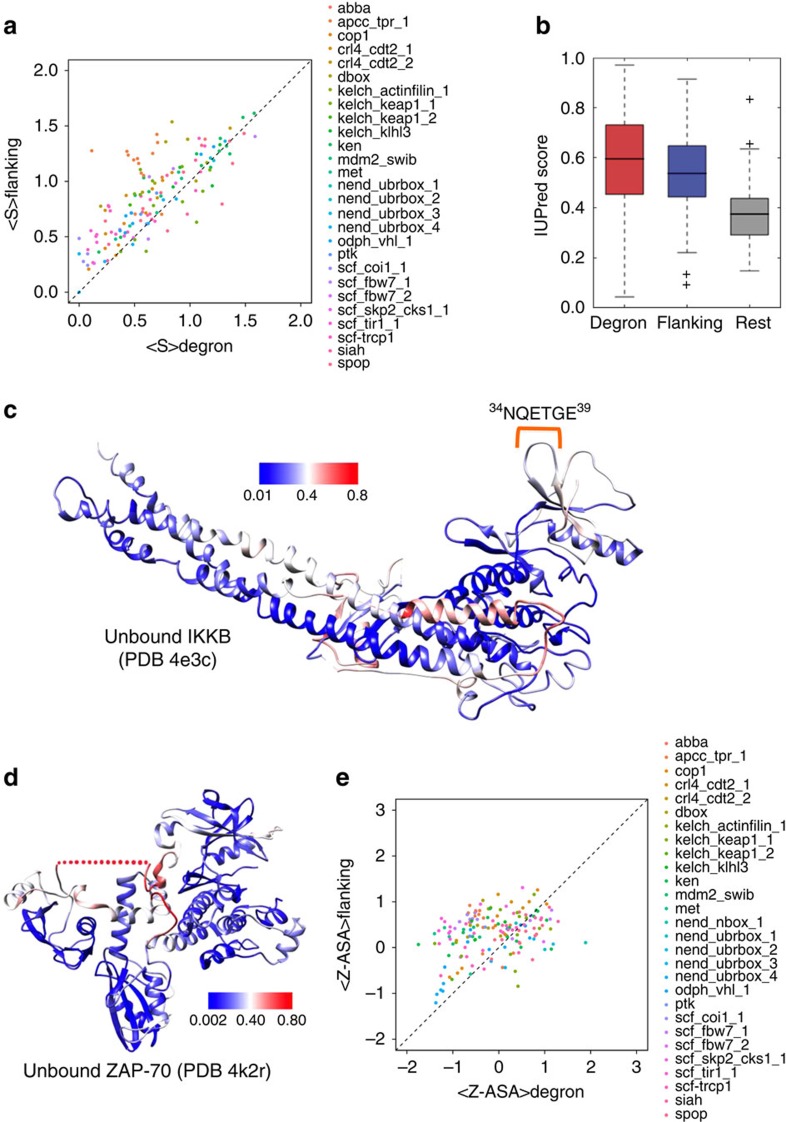
Characteristic features of primary degrons. (**a**) Plot of the average sequence entropy of primary degron sequences (<S>degron) versus their flanking residues (<S>flanking) for proteins belonging to the primary degron data set. Points above the diagonal indicate proteins, for which <S>degron is smaller than <S>flanking, that is, the degron sequence is more conserved in comparison with the degron-flanking sequences. (**b**) Box plot showing the average IUPred disorder scores of primary degron residues, flanking residues and the remaining protein sequences (for all the primary degron instances). IUPred scores of 0.5 and above indicate disordered residues. (**c**) Protein Data Bank (PDB) structure of the inhibitor of nuclear factor-κB kinase subunitβ, IKKB (PDBid: 4e3c) with the primary degron indicated. The residues are coloured according to IUPred disorder scores (colour scale is shown, red indicates disordered residues). (**d**) PDB structure of unbound ZAP-70, an essential tyrosine kinase important in immune response (PDBid: 4k2r). In the structure, a 45-residue segment containing the 7-residue degron is missing from the electron density (red dots). The residues are coloured according to IUPred disorder scores (colour scale is shown). (**e**) Average ASA *Z*-scores for primary degrons versus their flanking residues (see Methods). Points lying above the diagonal indicate proteins for which the average Z-ASA for the degron segment is lower than that of the flanking regions. Flanking regions in **a**,**b** and **e** include ten residues on the N-terminal and ten residues on the C-terminal side neighbouring the primary degron.

E3 ligases target specific substrates for Deg by recognizing their primary degron (Fig. 1a). We catalogued 28 primary degron motifs encompassing a broad functional range, from 171 experimentally validated instances in 157 diverse substrates (Table 1 and Supplementary Tables 1 and 2). Analysis of their properties revealed that primary degrons resemble typical SLiMs. SLiMs are short (typically 5–10 residues), evolutionarily conserved functional peptide segments present within IDRs that mediate interactions with partner proteins^16^. Functional regions (such as binding motifs) behave as islands of sequence conservation within fast diverging IDRs. As E3 ligases bind substrates via their primary degrons, as part of a serious decision on the protein's fate, the functional importance of degrons across orthologues is clearly reflected in their highly significant sequence conservation (Fig. 2a).

In terms of their structural preferences, primary degrons tend to be located within segments that are predicted to be intrinsically disordered (IUPred^17^, Fig. 2b), with high local backbone flexibility (DynaMine^18^, Supplementary Fig. 2), as compared with the overall substrate sequences in which they occur (*P*<2.2E−16). Again, this mirrors the general tendency towards local structural disorder for functional SLiMs. Consistently, we found that a large majority is located outside domain boundaries: 79% of primary degron instances had 0% overlap with annotated Pfam domains^19^ and the remainder localized to surface loops of structured domains (Fig. 2c). Secondary structure propensities calculated using PSIPRED^20^ also indicated a predominance of coil conformations (∼85%) in degrons.

We also searched the Protein Data Bank^21^ for unbound structures of all 157 substrates, to analyse the primary degron before E3 recognition. Although we found 505 unbound structures (corresponding to 65 substrates), in the majority of cases the degron-containing segment was not part of the experimental constructs used, most probably because of its location outside domains—in a potentially disordered region—which hampers crystallization. Only in 17 structures (8 substrates) the primary degron sequence was part of the experimental construct used (Supplementary Table 3) and only for one protein the degron region was actually visible in the structure (Fig. 2c). For the remaining structures the degron regions have missing electron density (one such example is shown in Fig. 2d) and they fall into disordered regions. In contrast to the unbound structures, many primary degrons undergo disorder-to-order transitions when they bind to cognate E3 ligases (Supplementary Table 4), indicating conformational stabilization on binding (Supplementary Fig. 3); such behaviour is typical of motif-mediated interactions serving to impart recognition specificity^16^.

### Surface accessibility of primary degrons infers regulation

It is clear from analysis presented in the previous section that the majority of degrons are present in locally disordered regions. However, to investigate the possibility that subtle, local modulations in surface accessibility may regulate degron recognition by E3s, we investigated the surface accessibility of primary degrons relative to their flanking residues. Because of missing structure coordinates (both of degrons and degron-flanking sequences), we predicted accessible surface areas (ASAs) from sequence using SPINE-X^22^ (see Methods for details). Per-residue absolute ASA values were converted into *Z*-scores (using amino acid type-specific ASA distributions, shown in Supplementary Fig. 4, to enable comparisons between regions comprising different residue types) and then the average *Z*-score was computed for both the degron and degron-flanking segments in each of the 157 proteins. We observed a statistically significant trend for primary degrons to have a lower average Z-ASA as compared with their flanking regions (<Z-ASA>_degron_<<Z-ASA>_flanking_, *P*=8.3E−12; Fig. 2e). This leads us to speculate on the existence of conformations where the degron may be locally buried/shielded by its neighbourhood (for example, by steric occlusion) and thereby held in conformations that are unsuitable/inaccessible for recognition by E3s.

Dissected by degron type (Supplementary Fig. 5), only the N-terminal (N-end rule) degrons are more exposed than their flanking residues; for the majority of the remaining categories the primary degron has a lower average Z-ASA compared with its flanking regions (significant differences were observed in case of APC/C (DBOX), APCC_TPR_1, CBL (MET), COP1, CRL4_CDT2_1, KLHL3, MDM2_SWIB, SCF_COI1_1, SCF_TIR1_1 degrons and the SCF_SKP2-CKS1_1 and SCF_TRCP1 phospho-degrons). This observation potentially indicates regulatory control that couples signalling events with degron accessibility (via local conformational changes, as we discuss in the following section).

### PTMs regulate degron accessibility and conformational state

By keeping the primary degron relatively occluded compared with its flanking regions and thus inaccessible to recognition by E3 ligases, Deg can be made conditional and subject to regulation. Given the extensive cross-talk between phosphorylation and ubiquitination^23^, a putative mechanism may involve the initiation of Deg via an appropriate (priming) PTM in the degron neighbourhood resulting in changes in degron accessibility by shifting the local conformational equilibrium towards more exposed states of the degron. Indeed, degron-flanking phosphorylation events that induce a conformational change resulting in degron exposure have been proposed in the Deg of Chk1 (ref. 24), Cdc25A and Cdc25B phosphatases^25^,^26^. In a recent molecular dynamics study, it was also shown that phosphorylation of a disordered segment shifts the conformational equilibrium towards binding competent sub-states^27^.

In several substrates, multiple (sequential) priming modifications are essential for Deg to occur. For example, coordinated phosphorylation by multiple kinases occurs in the inhibition of the transcriptional coactivator Yes-associated protein, a human oncoprotein^28^. Phosphorylation of the ^376^HXRXX**pS**^381^ motif by the Lats tumour suppressor provides the priming signal for CK1δ/ɛ to phosphorylate the neighbouring ^383^D**pS**GX**pS**^387^ phosphodegron in Yes-associated protein and enables recognition by β-TRCP, the substrate recognition subunit of SCF^β-TRCP^. In case of β-catenin, casein kinase Iα primes Ser45, causing relay phosphorylation of Thr41 by glycogen synthase kinase 3, followed by Ser37 and Ser33 creating a canonical D**pS**GXX**pS** phosphodegron^29^. A priming phosphorylation is also required for the proteolytic turnover of interferon receptor (IFNAR1)^30^. In the light of our ASA calculations, these events can be hypothesized to occur via local conformational shifts that enable formation of binding-competent sub-states that are accessible/compatible for E3 binding. Certain IDR amino acid compositional types (polar tracts and polyelectrolytes^31^) possess a tendency to form nonspecific and unstructured, but locally compact, conformations (which can maintain the degrons in a buried/sterically occluded state); this process can be reversed by increasing the net charge per residue by the addition of PTMs such as phosphorylation. The fact that most known degrons are present within locally disordered regions would make PTM-mediated structural shifts highly sensitive and lend itself to fine-tuning of the regulation of Deg.

Perhaps reflecting the generality of this mechanism, we found 121 experimentally annotated PTM sites corresponding to 48 primary degrons (from 45 proteins) in our data set. Eighty-four of these PTM sites were located in flanking regions (considering upto 20 residues upstream and downstream of the degron in the primary sequence; Supplementary Data 1). Furthermore, mutations in many of these flanking residues that are known to be modified by PTMs lead to reduced Deg rates and loss of E3 binding (Supplementary Data 2), which clearly demonstrates the mechanistic importance of the nearby PTM event in priming primary degron recognition by E3 ligases. The potential for PTM (for example, phosphorylation)-based regulation of degron accessibility (and conformational state) is also indicated by the high incidence of Ser, Thr and Tyr residues (∼19%) in the degron-flanking regions in these 157 proteins. These residues, many of which show a high sequence conservation among related proteins (Supplementary Fig. 6), can potentially be modified by phosphorylation.

In addition to phosphorylation, there is evidence for a dynamic regulatory interplay between *O*-GlcNAcylation at Ser/Thr sites and protein stability: for example, *O*-GlcNAcylation of p53 at Ser-149 prevents the phosphorylation of adjacent Thr-155, which blocks ubiquitin-dependent proteolysis and stabilizes p53 (ref. 32). Thus, there is speculation that *O*-GlcNAc could act as a protective signal against proteasomal Deg, perhaps by counteracting the effect of phosphorylation^33^.

### Secondary degrons are Deg linked substrate Ubsites

The selection of primary degrons is followed by the ubiquitination (poly-, mono- or multiple monoubiquitination) of acceptor Lys residue(s) on the substrate by the E3–E2 machinery (Fig. 1). The complexities of ubiquitin chain linkages and their functional readout is a field of intense study^6^. We define the secondary degron as the substrate lysine(s) that are ubiquitinated and linked to Deg. Although a Lys48-linked tetra-ubiquitin chain was widely believed to be the canonical/minimal determinant for proteasomal Deg, there is increasing evidence for other chain lengths and also (multiple) mono-Ub moieties to serve as efficient proteasomal targeting signals^34^. Here we have analysed two data sets (details in Methods) as follows: (i) 108 Deg-linked lysines on 42 proteins (‘Deg') and, as control, their remaining lysines (‘Others'); and, ii) 9,323 ubiquitinated lysines in 3,756 proteins (ubiquitination sites (‘Ubsites')), based on two proteomics studies^35^,^36^, however, without annotation as to the outcome of ubiquitination. Their control set was an equivalent number of non-ubiquitinated lysines from the same set of proteins (‘Non-Ubsites'; Supplementary Data 3).

### Secondary degrons are characterized by structural flexibility

**Figure 3:**
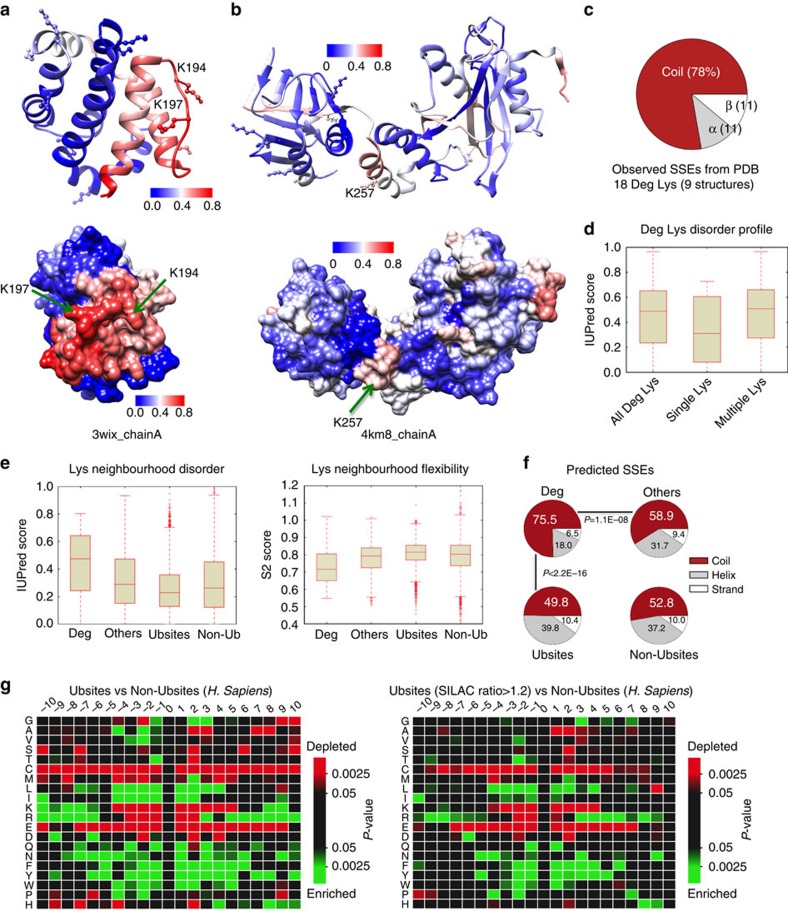
Features of secondary degrons and their sequence neighbourhood. Protein Data Bank (PDB) structures of (**a**) induced myeloid leukemia cell differentiation protein Mcl-1 (PDBid: 3wix) and (**b**) suppressor of fused homologue SUFUH (PDBid: 4km8) coloured by IUPred residue-wise disorder scores (colour scales shown, red indicates highly disordered). All lysine residues are shown as ball-and-stick models (Deg lysines additionally indicated by text and arrows). (**c**) Observed SSE distribution of 18 (out of 108 Deg lysines) present in the nine PDB structures of eight proteins from the Deg data set (Supplementary Table 5). (**d**) Box plot of the IUPred disorder scores of Deg lysines (‘All Deg Lys', the complete set of 108 Deg lysines; ‘Multiple Lys', Deg lysines from proteins carrying multiple Deg Lys; ‘Single Lys', Deg lysines from proteins that contain a single Deg Lys). IUPred scores of 0.5 and greater indicate disordered residues. (**e**) IUPred disorder scores (left) and local flexibility (DynaMine S2 score, right) calculated using a 21-residue window centred on lysines from each group. DynaMine scores <0.7 indicate residues with high local backbone flexibility, values between 0.7 and 0.8 indicate context-dependent flexibility and values >0.8 indicate well-structured residues. (**f**) Secondary structure propensities of residues in the 21-residue window around the different lysine groups. The *P*-values are from *χ*^2^-tests. (**g**) Heatmaps showing enrichment or depletion of the 20 aa types in the neighbourhood of ubiquitinated lysines (position 0) from Ubsites (left) and the subset of Ubsites that showed quantitative increase in ubiquitination on proteasomal inhibition (right). The comparison is always relative to Non-Ubsites. The heatmaps were created using iceLogo^68^.

In an early paper, Varshavsky^37^ suggested a ‘stochastic capture' model for the ubiquitination of N-end rule substrates, suggesting that the probability of a Lys to serve as the Ubsite was proportional to its local flexibility. The rationale was that too rigid a conformation would reduce ubiquitination efficiency and render it incompatible with processive polyubiquitination. Recent bioinformatics analyses have led to somewhat opposing results claiming that Ubsites do^38^ or do not^39^ fall into locally disordered regions, although Deg-linked Ubsites were clearly more disordered^39^. We addressed these features by analysing both available structural data and disorder predictions of the lysine data sets. Only for 8 of the 42 Deg substrates, we found 9 non-redundant Protein Data Bank structures (in which only 18 of the 108 Deg Lys were visible; see Supplementary Table 5). Two structures (possessing 2 and 1 characterized Deg lysines) are shown in Fig. 3a,b. In both cases, even though structured in the crystal environment, the surface patch (ubiquitination zone^10^) containing the Deg lysine(s) were predicted to be the most disordered/flexible. From this limited structural data for the 18 Deg lysines, we also plotted their observed secondary structural elements (SSE) distribution (Fig. 3c). Non-regular (coil) conformations are highly preferred (∼80%), mirroring previous analysis of *in vivo* Ubsites that showed a majority of lysines to be present on surface loops, followed by α-helices^39^,^40^.

Based on predictions of structural disorder (IUPred) for the complete Deg data set, we observed that about half of the Deg lysines are in IDRs (median IUPred disorder score ∼0.5); interestingly, Deg regions containing multiple Deg-linked lysines tend to have higher disorder scores than those regions with single Deg lysines (*P*<0.05; Fig. 3d). This suggests a greater role of structural plasticity for ubiquitination of redundant neighbouring lysines and this is also demonstrated by the two previous examples: the ubiquitination surface containing multiple Deg lysines (Fig. 3a) is predicted as being more disordered than the surface with a single Deg lysine (Fig. 3b).

Using disorder predictions (IUPred) and predictions of local backbone flexibility (DynaMine), we also analysed the neighbourhood of the lysines (using 21-residue sequence windows centred on each lysine). Deg lysine neighbourhoods are significantly more disordered than the other lysine categories (Deg versus Others, *P*=2.8E−7; Deg versus Ubsites, *P*<2.2E−16; and Deg versus Non-Ubsites, *P*<2.2E−16; Fig. 3e, left). Similarly, Deg neighbourhoods are characterized by significantly higher backbone flexibility (lower DynaMine S2 scores; Deg versus Others, *P*=5.1E−8; Deg versus Ubsites, *P*<2.2E−16; and Deg versus Non-Ubsites, *P*=1.3E−10; Fig. 3e, right). These observations correlate with predicted secondary structural propensities: the majority (>75%) of Deg lysines occupy coil conformations and this preference is significantly higher than all the other categories (Fig. 3f). Thus, an environment characterized by significantly increased local disorder/flexibility appears to specify Deg-linked Ubsites. We surmise that stable anchoring of the substrate to the E3 ligase via the primary degron combined with structural adaptability around the ubiquitination surface enables processivity in ubiquitination and the choice of multiple lysines to modify^41^.

### Sequence features neighbouring the secondary degron

Unlike for primary degrons, no general sequence motif(s) encompassing the secondary degron have been established. Ubiquitin transfer occurs in a special microenvironment created by E2 active site residues, the acceptor Lys on the substrate and its neighbouring residues. Therefore, the lack of globally identifiable motif preferences could be due to E2-specific catalytic mechanisms^42^,^43^ that necessitate a requirement for compatibility between the amino acid environment of the acceptor lysine and key residues within the E2 catalytic core, as demonstrated for yeast Cdc34 (ref. 44).

We analysed amino acid frequencies within a 21-residue window centred on Ubsites lysines (enrichment calculated relative to the Non-Ubsites set, Fig. 3g). Aromatic and hydrophobic residues were significantly over-represented (in particular within ±6 residues of the lysine), whereas Cys, Met and charged residues are depleted (Fig. 3g, left). Whereas Glu is disfavoured throughout, the positively charged residues (Arg and Lys) are depleted in the immediate vicinity but enriched further away (>6 aa). Although the general trend is towards the depletion of strongly helix-favouring residues (Arg, Lys, Glu and Met), Leu and Gln appear exceptional, as they are enriched at certain positions. Asn is also enriched; however, it does not possess striking propensities for forming any regular secondary structure elements. Further, Gly and Pro are also over-represented at certain positions in the vicinity of Ubsites compared with Non-Ubsites. In one of the two proteomics data sets included in Ubsites, putative Deg-linked sites were ascertained using a proteasomal inhibitor and SILAC (stable isotope labelling by amino acids in cell culture) strategy^35^. We compared the residue usage in this subset with substantially increased ubiquitination after proteasome inhibition (SILAC ratio>1.2), putatively linked to Deg. However, residue frequencies were qualitatively very similar (Fig. 3g, right). Finally, we also checked the residue composition for Deg versus Others. Although the number of Deg lysines is considerably less than the number in Ubsites (making a strong statistical trend less probable), nevertheless the enrichment of aromatics is still evident (Supplementary Fig. 7). Interestingly, Thr and Tyr are also enriched around Deg sites, suggesting a possible role for PTMs in secondary degron selection. A previous study also observed significant enrichment of phosphorylatable residues (Ser, Thr and Tyr) flanking *in-vivo* Ubsites^40^.

Although overall residue usage preferences were evident (Fig. 3g), we were unable to determine any specific (enriched) sequence motifs. Nevertheless, the neighbourhood of ubiquitinated lysines is clearly important, forming an additional layer of regulatory control, as several studies on specific E2s have demonstrated the dependence of lysine selection on specific local amino acid preferences. For example, efficient Ub chain initiation by the APC/C-specific E2 Ube2C is dependent on charged residues close to the preferred Lys, which determine the timing and rate at which substrates are degraded by the APC/C^45^. Substrate lysines selected by the yeast E2 Ubc4 contain neighbouring acidic residues that are complementary to a highly conserved Lys (K91) adjacent to the catalytic Cys86 of Ubc4 (ref. 46). Ubc4 is a specialized ‘initiator' E2: yeast APC/C uses Ubc4 for chain initiation and Ubc1 for K48-linked chain elongation^47^. K48 selectivity of Ubc1 arises from a cluster of polar residues proximal to the Ubc1 active site^42^. Cdc34 is another E2 that displays specificity for Ub-K48 and this behaviour is promoted by the presence of an acidic loop near the E2 active site that optimally positions ubiquitin K48 for nucleophilic attack^48^. These results suggest that different E2s use distinct strategies to determine acceptor sites and detailed studies attempting to determine any corresponding specificity determinants (ubiquitination motifs) should consider E2 specificity.

### Relationship between primary and secondary degrons

Once the active E3–E2/substrate assembly has formed, spatial and geometric constraints such as distance and orientation relative to the E3-bound primary degron^49^ limit the ubiquitination surface and the selection of Deg-linked lysine(s). Thus, the relative separation between primary and secondary degrons should be an important and conserved feature. Indeed, altering the distance between the Dbox primary degron and Ub-initiation motifs in the APC/C substrate geminin stabilizes the protein against Deg^45^.

Although we do not have sufficient data to draw general conclusions, for 11 proteins present in both the primary degron and Deg data sets, we could investigate the positions of Deg lysines relative to the primary degron (Supplementary Fig. 8). P53 has 17 characterized Deg lysines and relative to its N-terminal MDM2-binding site, the lysines fall into multiple distance bins (Supplementary Fig. 8A). For most of the remaining proteins, however, we observed a clear distinction between Deg and Other lysines: the former (Deg) tend to be located very close in sequence to the primary degron (often within 20 residues). Pavletich and colleagues^49^ have commented on the fact that β-catenin (Supplementary Fig. 8C) and IκBα (Supplementary Fig. 8D) orthologues and paralogues all contain lysines located 9–14 residues upstream of their primary degrons. Further, they showed that altering the relative spacing between the primary and secondary degron sites in β-catenin strongly influenced ubiquitination efficiency.

This apparent proximity of the primary (E3-binding motif) and secondary degron (site(s) of ubiquitination) suggests another possible role for the Ser/Thr residues flanking the primary degron (described earlier). Although ubiquitination occurs mostly on Lys residues, evidence exists that Ser/Thr can also be modified by ubiquitin or Ub-like proteins, thus replacing the Lys as a secondary degron site in proteins such as BH3 interacting-domain death agonist, neurogenin and the heavy chain of major histocompatibility complex I^34^.

Physical separation thus uncouples E3-binding and ubiquitination but also should enable a degree of allosteric control between these functionalities. Thus, it is possible that E3 binding to the primary degron increases reactivity of Lys at the secondary site. Each lysine will possess a distinct probability of being the secondary degron, with proximity to the primary degron being a strong determinant in most cases (Supplementary Fig. 8). Further factors such as local structural flexibility and sequence neighbourhood (Fig. 3) also strongly contribute to defining a unique kinetic code of modification. For example, ubiquitination of yeast Sic1 (after binding to SCF^Cdc4^) is restricted to six lysines in the N-terminal domain and each modification seems to have a different readout in terms of Deg efficiency and downstream signalling^50^.

### The tertiary degron initiates substrate unfolding

Recent evidence suggests that (poly)ubiquitination may not be sufficient for efficient proteasomal Deg, which additionally requires a disordered (or partially unfolded) region on the substrate^7^,^9^. The proteasome initially engages with this flexible segment (tertiary degron) and the substrate is then unfolded in a cooperative, ATP-dependent manner (Fig. 1c,d). Based on recent structural details of the proteasome^8^, the ubiquitin receptors Rpn10 and Rpn13 on the 19S regulatory particle are located ∼70–80 Å away from the ATPase unfolding channel. To facilitate access to these buried sites and thereby entry into the catalytic core of the 20S particle, the tertiary degron apparently requires a minimal length of 20–30 residues and needs to be located adjacent to the polyubiquitin tag. Deg efficiency drops sharply when the two sites are gradually separated^9^.

**Figure 4:**
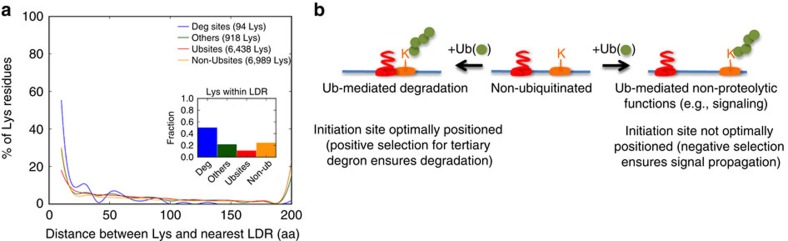
Sequence distance between secondary and tertiary degrons. (**a**) Plot showing the percentages of lysine residues (from each of the four data sets: Deg, Others, Ubsites and Non-Ubsites) that are within a given distance (number of amino acids away) from the nearest LDR (see Methods) predicted using IUPred. The inset shows the fraction of lysines that are located within an LDR. (**b**) Schematic outline of the distance preferences between secondary and tertiary degrons for ubiquitinated, Deg-linked lysines and lysines with regulatory (non-proteolytic) functions following ubiquitination.

We therefore investigated the presence of long disordered regions/segments (LDRs; defined as at least 20 consecutive disordered residues, see Methods) in the vicinity of known Ubsites in physiological substrates. IUPred calculations show a strong distinction in this feature between Deg and all the other lysine categories: nearly 60% of Deg sites are located in the proximity (within 0–10 residues) of an LDR as compared with only 20–30% in the other Lys categories (Fig. 4a). DynaMine predictions also show that a significantly higher fraction (∼75%) of Deg lysines possess a flexible segment less than ten amino acids away (Supplementary Fig. 9). Considering the fraction of sites located within LDRs (Fig. 4a inset and Supplementary Fig. 9 inset), it is significantly more for Deg lysines than for all the other categories. Not only do these results confirm the earlier observations that Deg sites are significantly more disordered/flexible among all lysine categories (Fig. 3e,f) but it indicates a strong correlation between substrate Deg and the requirement for a proximal disordered Deg initiation site.

Thus, a disordered region of adequate length for proteasomal entry appears to be an essential component of the signal for Deg. Furthermore, transient local unfolding enabled by intrinsic flexibility or promoted allosterically by external factors (for example, binding of AAA-ATPase p97 of ‘unfoldase' activity^51^) or posttranslational modifications (for example, phosphorylation of the CDK inhibitor p19^4inkd^ (ref. 52)) can also be used. Further, polyubiquitination itself can promote unfolding and exposure of the tertiary site in a ‘regulated unfolding' mechanism^53^,^54^.

### Local disorder and outcome of ubiquitination

Relative local disorder/flexibility can putatively distinguish ubiquitinated lysine sites that are connected either with Deg or regulation-linked events. The fraction of Ubsites located within LDRs (10%) is significantly lower than the equivalent fraction not only of Deg sites (50%) but remarkably also of non-ubiquitinated control sites (that is, Non-Ubsites and Others, with 24% and 21% respectively; Fig. 4a inset), which suggests that many of the identified Ubsites may not be linked to protein Deg. This completely agrees with the observation that ∼40% Ubsites did not show any significant increase in site-specific ubiquitination on proteasomal inhibition^35^. Thus, the observation (that is, presence/absence) of local structural disorder might be useful to predict the functional outcome of protein ubiquitination and it may also have evolutionary implications. The comparison between Ubsites specifically linked to Deg and all Ubsites relative to the two non-ubiquitinated control sets (Fig. 4) suggests that the distance between the Ubsite and the nearest LDR is likely to be under strong evolutionary pressures such that Deg-linked sites show positive selection in favour of overlapping disordered regions, whereas sites involved in signalling exhibit negative selection to remove proximal LDRs to prevent Deg of the protein, or at least reduce its efficiency (Fig. 4b). The importance of the tertiary degron is especially striking, as variations in this component appear to significantly impact protein half-life and many paralogues affected by such changes (those with significantly shorter flexible segments) were observed to possess signalling functions^55^.

### Degron components and disease links

**Figure 5:**
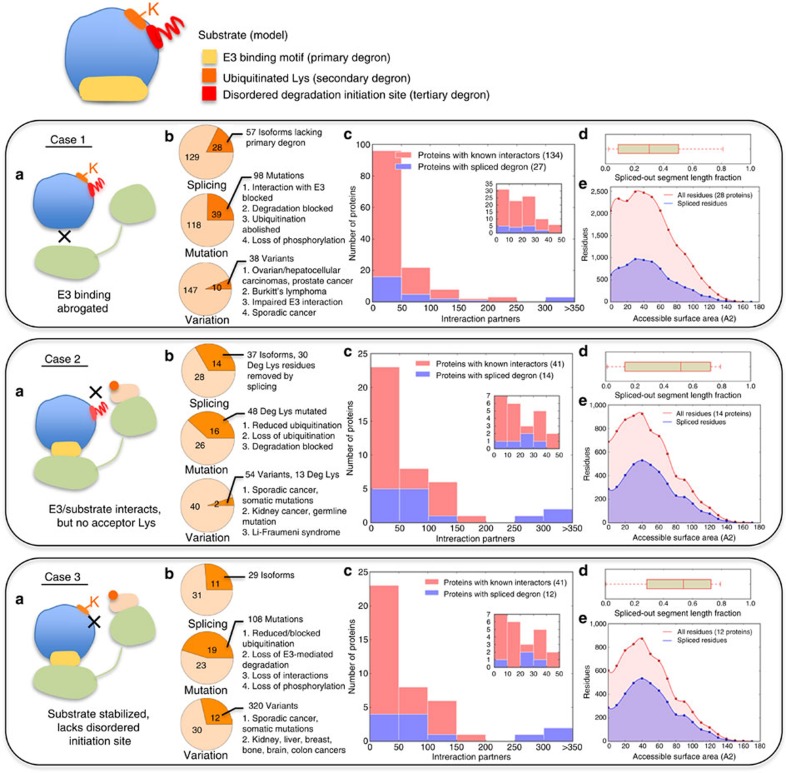
Tripartite degron components and functional impacts on their abrogation. Large-scale data on alternative isoforms, mutations and sequence variations affecting the primary (case 1), secondary (case 2) and tertiary degrons (case 3). Case 1 corresponds to the 157 proteins in the primary degron data set (Supplementary Table 2), case 2 corresponds to the 42 proteins in the Deg data set (Supplementary Data 3) and case 3 refers to LDRs (predicted using IUPred) that are located nearest to the Deg lysines (that is, the tertiary degrons). For proteins in which Deg lysine(s) were located inside the tertiary degron, the mutation/variation data for the latter (case 3) include changes at the lysine(s). (**a**) Schematic overview of the consequences of missing (or corrupted) degrons. The same colour scheme is used as in Fig. 1. (**b**) Pie charts showing the number of proteins (out of the total number in each data set) for which alternative isoforms with missing degrons (top), mutations (middle) and variations (bottom) of degron components have been experimentally characterized (orange), the number of corresponding isoforms/mutants/variants and a brief description of their major functional impacts and/or associated disease outcomes (full details are provided in Supplementary Data 4–6). (**c**) Histograms showing the numbers of proteins that have between 0 and 50, 51 and 100, 101 and 150, and so on validated interaction partners (pink bars). Overlaid in blue are the numbers of proteins from each interaction bin that possess characterized isoforms in which degron components have been removed by alternative splicing. The insets are used to show in finer detail the bin corresponding to 0–50 interaction partners. (**d**) Box plot showing the length fraction (as compared with the canonical sequence) of protein segments removed by alternative splicing. (**e**) Plot of ASA distribution for those proteins where degron components are removed by alternate splicing. ASAs of all residues from the canonical sequence are shown in light red, whereas ASAs of residues that are removed in one or more isoforms are in blue. ASA values were predicted with SPINE-X^22^ using the canonical sequences as input.

To further the evidence for the validity and the functional importance of the tripartite model, we next examined how impairing degron elements is linked to disease. We reasoned that a corrupted primary degron should abrogate substrate targeting by E3 ligases, a mutated secondary degron should block substrate ubiquitination (unless the lysine selection for a particular substrate is less stringent, as in the case of multiple neighbouring redundant lysines) and finally removal of the tertiary degron or mutations (for example, the removal of phosphorylatable residues) should alter substrate Deg kinetics (Fig. 5a). For the proteins in our primary degron and Deg data sets, UniProt annotations were scanned for experimental evidence of modifications (for example, isoforms resulting from alternative splicing, natural sequence variants including polymorphisms and disease-associated mutations) that interfere with the known degron components (see Methods). The results are summarized in Fig. 5b: in many cases, degron abrogation is directly linked with diseases such as cancer and growth defects (Supplementary Data 4–6). As mutations affecting degron elements are likely to critically influence cellular physiology, our model suggests that many more disease links are yet to be discovered.

### Interfering with degrons also rewires the local interactome

As modifications affecting many of the known degron components have profound effects on cell physiology (as manifested in causing disease; Fig. 5b), we inferred that many of these proteins with deleted degron component(s) may take central positions in the local interactome, that is, they are highly connected. In fact, based on data from experimentally validated interactomes (Methods), many of these proteins are hubs (57% and 68% of proteins from the primary degron and Deg data sets, respectively, have >20 experimentally validated binding partners). After binning the proteins according to their number of known interaction partners (Fig. 5c), we observed that several proteins for which alternative splicing causes the removal of known degron component(s) are present in high interaction density bins. Similar observations are likely to grow as experimental evidence about protein isoforms and data on degron components become increasingly available. Here we discuss protein isoforms (often resulting from alternative splicing), because this may result in local interactome rewiring and affect protein function in complicated ways: first, the increased protein availability (degron removal often increases protein half-life by orders of magnitude) would affect equilibrium concentrations of different protein complexes; second, alternative splicing removes significant portions of the protein sequence (Fig. 5d) and affects considerable parts of the exposed surface (Fig. 5e), thus altering the interaction landscape by removing interaction patches/sites for certain partners.

**Figure 6:**
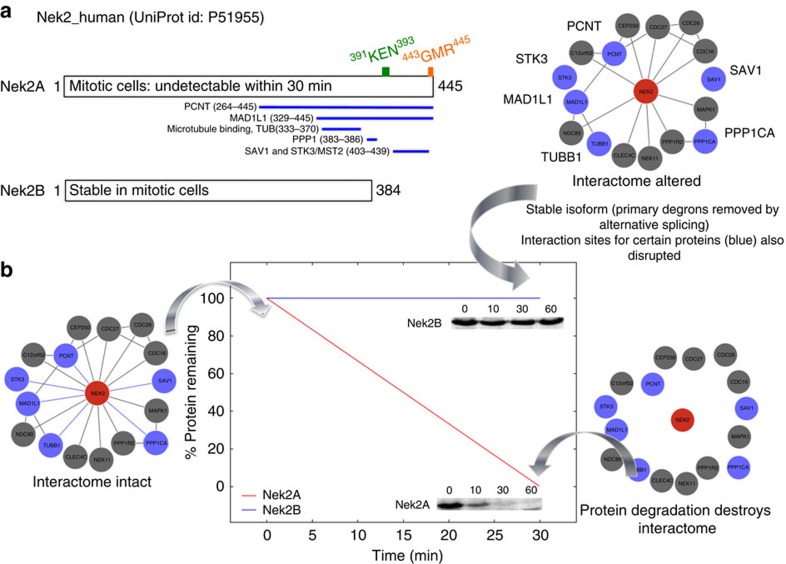
Removal of primary degrons influences protein stability and interactions. (**a**) Specific example of human NIMA-related kinase 2 (Nek2) (UniProt ID: P51955) showing the canonical sequence (Nek2A, top) and a shorter splice form (Nek2B, bottom) with two primary degrons missing from the C-terminal region. Sequence regions on Nek2A that are responsible for protein–protein interactions with partner proteins are marked with blue lines (data from UniProt). (**b**) Computed plot (middle) of the amounts of Nek2A (red) and Nek2B (blue) isoforms remaining as a function of time (data from ref. 57). The gels are from *in vitro* Deg assays performed by adding ^35^S-labelled Nek2A and Nek2B to CSF extracts with addition of calcium (to trigger anaphase entry) (adapted from ref. 57 with permission). The local interactome of canonical Nek2 (red circle) is drawn showing experimentally validated protein partners: blue circles represent proteins whose binding is affected by alternative splicing (missing lines indicate disrupted interactions).

The possible complex outcome is illustrated using the example of NIMA-related kinase 2 (Nek2; Fig. 6). Nek2 is a serine/threonine protein kinase that regulates centrosome separation in mitotic cells and controls chromatin condensation in meiotic cells. Two (primary) degrons have been characterized in the non-catalytic, carboxy-terminal domain of Nek2A (a KEN-box and an exposed C-terminal MR dipeptide tail)^56^. Alternative splicing of this protein removes these two primary degrons and the resulting isoform (Nek2B) is significantly more stable (Fig. 6a)^56^,^57^. In addition, this splicing event also removes the interaction regions of several binding partners (Fig. 6a), thus initiating a complex remodelling of the local interactome, realizing at least three distinct cellular states (Fig. 6b) involving Nek2A (following synthesis), Nek2A (mostly degraded after 30 min) and Nek2B (stable but having a reduced/altered interaction capacity). Protein availability and functional lifetime are crucial parameters, as for example; elevated Nek2 levels have been detected in a number of human cancer types and cancer-derived cell lines^58^. In addition to stability changes due to degron removal (or mutations), the subset of disrupted interactions will also be critical. For instance the C terminus of Nek2A, which is missing in Nek2B, contains a binding site for the catalytic subunit of protein phosphatase 1 (Fig. 6b), which acts as a physiological inhibitor of Nek2 (ref. 59). Thus, interactome rewiring by changes in protein stability (due to degron removal) and alterations in the interaction landscape are likely to be a common biological phenomena that would help to understand disease pathways.

## Discussion

Regulated Deg is a fundamental mechanism used to exert control over cellular processes and pathways by enabling precise alterations in protein levels. The distributed nature and synergistic relationships between the E3 recognition/docking site on the substrate (primary degron), Ubsite (s) (secondary degron) and proteasomal Deg initiation site (tertiary degron) (Fig. 1) will help explain the diversity and specificity of the ‘degrome'. However, it also infers new challenges for the identification of degron components and delineating their regulatory relations. What is clearly apparent however is that the multipartite nature of degrons also maximizes the regulatory complexity in decision-making before removing a protein from the cell. Another challenge for the structural characterization of degrons will arise from their presence within intrinsically disordered protein regions (Figs 2, 3, 4). The importance of conformational dynamics in degron functionality and regulation via PTMs will also need to be carefully studied further. Degron elements can also fundamentally control biological functioning as altered half-life due to the removal/mutation of degron elements will have a significant impact on the duration of activity of a protein. In addition, alternative isoforms with missing degrons will not only have altered stability, but also affected interaction capacity, thus enabling a complex and temporal rewiring of the interactome (Figs 5 and 6).

## Methods

### Prediction of structural disorder and backbone flexibility

IUPred^17^ and DynaMine^18^ software were used to predict structural disorder and protein backbone dynamics, respectively, using amino acid sequences as input. IUPred outputs scores (ranging between 0 and 1) for each residue; scores >0.5 indicate disordered residues (the ‘short' mode of IUPred was used). DynaMine, trained on NMR data, estimates protein backbone dynamics and outputs per-residue S2 order parameter values, indicating residue flexibility. Values ≤0.69 indicate highly flexible residues; [0.7–0.8] shows context-dependent flexibility and >0.8 indicates ordered residues. Using the IUPred scores, LDRs were defined as consecutive stretches of at least 20 disordered residues (breaks of upto three consecutive ordered residues within an LDR were permitted).

### Prediction of secondary structure propensities

Secondary structure propensities were calculated from primary sequence using the PSIPRED software^20^ using default settings. The output provides for each residue a classification: C (coil), H (helix) or E (strand).

### Prediction of residue surface accessibility

SPINE-X achieves accuracy >80% in predictions of ASAs based on amino acid sequences as input^22^. SPINE-X outputs ASA values for every residue in the input sequence. Absolute ASAs were converted into *Z*-scores, to facilitate comparison of relative solvent accessibility between regions consisting of different residue types. The protocol used was as follows: using SPINE-X predictions for all 157 proteins in our primary degron data set, we built ASA distributions for each of the 20 amino acid types (Supplementary Fig. 4). Next, for a specific motif instance, the absolute ASA of each motif residue was converted into a *Z*-score (using the ASA distribution corresponding to its amino acid type) and the average *Z*-score calculated for that motif. The same protocol was followed when estimating the average *Z*-score for motif-flanking regions.

### Orthologue alignments and calculation of sequence conservation

Pre-computed multiple sequence alignments of orthologues were obtained from Discovery@Bioware (http://bioware.ucd.ie/~compass/biowareweb/) and used to calculate Shannon entropy scores for each aligned position ‘*i*' using the equation: *S*(*i*)=−Σ*p*(*k*).ln(*p*(*k*)), where *p*(*k*) is the probability of the *i*th position in the sequence alignment being occupied by a residue of class ‘*k*'. The classifications of residues used were as follows: [(Ala, Val, Leu, Ile, Met, Cys), (Gly, Ser, Thr), (Asp, Glu), (Asn, Gln), (Arg, Lys), (Pro, Phe, Tyr, Trp), (His)]^60^. Substitutions within a group were considered conservative. The lower the sequence entropy at a given alignment position, the higher its evolutionary conservation. For a given region (for example, primary degron sequence), the sequence entropy values for each motif position, *S*(*i*), were calculated and then averaged (<*S*>_motif_=Σ*S*(*i*)/*n*_motif_, where *n*_motif_ is the number of motif residues).

### Data sets of ubiquitinated lysines

We used the following data sets of ubiquitinated lysines for analysis (Supplementary Data 3):

1. A set of 42 mammalian proteins where the ubiquitination of 108 lysines had been linked to their Deg (this set is referred to as Deg). This set had been compiled (and used in an earlier publication) based on the following criteria^39^: (a) all the Ubsites had been studied *in vivo*; (b) existence of literature and database (UniProt^61^, UbiProt^62^, Phosphosite^63^) evidence of ubiquitination, detected either by high-throughput mass spectrometry or by point mutations of specific lysines that abolish ubiquitination; (c) proteins for which the data quality precluded complete detection of Ubsites or proteins with ambiguous sites were excluded; and, importantly, (d) the 42 proteins have experimental evidence of undergoing UPS-mediated Deg (or processing) after ubiquitination. Deg lysines were compared with a control data set comprising the remaining 1,024 lysines (Others) from the same 42-protein set.
2. A large-scale set of experimentally validated Ubsites in human proteins had previously been collected and used to train a human-specific ubiquitin site predictor (hCKSAAP_UbSite)^64^. The outcome of ubiquitination was unknown for this set of proteins. This data had been compiled from two recent proteomics-scale assays (in which the Ubsites were assigned based on enrichment of endogenous ubiquitinated peptides using affinity purification followed by high-resolution mass spectrometry^35^,^36^) and from literature-derived UniProt annotations; the final set was prepared by filtering the proteins for sequence redundancy (using a 30% identity cutoff). This list was matched against the current UniProt release and contained 9,323 Ubsites (from 3,756 human proteins) that we refer to as Ubsites. For comparison, we used a set of 9,318 non-ubiquitinated lysines assembled from the same set of proteins (Non-Ubsites).

Sequence windows of 21 residues centred on the lysines were created for analyses of their features. In cases where the Lys was located near the termini of the protein chain, truncated sequence windows were used.

### Protein–protein interaction data

iRefWeb^65^ was used to retrieve data for known protein–protein interactions. The following filters were applied: (i) only physical interactions based on experimental validation; (ii) the interaction had been described in at least one publication; (iii) single-organism interactions only (that is, both proteins were from the same organism); and (iv) MI (MINT-inspired) score of 0.4 or more. This score is a measure of confidence in the observed interaction.

### UniProt annotations of sequence variants

Two data sets were used for cataloguing instances where degron elements were adversely affected: the primary degron data set of 157 proteins (Supplementary Table 2) and the Deg data set of 42 proteins (Supplementary Data 3). The Deg data set contains secondary degron instances (that is, Deg-linked lysines) and tertiary degrons were also defined using the same data set (that is, LDRs nearest to each Deg lysine). We searched UniProt annotations (feature tables, denoted ‘FT') for each of these proteins for cases where any of the degron components were missing/altered:

- Sequence variants (alternative sequence or isoforms, denoted ‘VAR_SEQ' in UniProt annotation) resulting from alternative splicing, alternative promoter usage, alternative initiation and ribosomal frameshifting; alternative splicing was the most abundant.
- Mutations (denoted ‘MUTAGEN') corresponding to site(s) that have been experimentally altered by mutagenesis and their effects studied.
- Sequence variations (position specific, denoted ‘VARIANT') as reported by authors; validated human polymorphisms are linked to entries in the Single Nucleotide Polymorphism database^66^. Entries in this category also include disease-associated mutations.

### Statistical tests

All statistical tests for calculating *P*-values were carried out using the Mann–Whitney *U*-test, unless otherwise specified.

## Additional information

**How to cite this article:** Guharoy, M. *et al*. Tripartite degrons confer diversity and specificity on regulated protein degradation in the ubiquitin-proteasome system. *Nat. Commun.* 7:10239 doi: 10.1038/ncomms10239 (2016).

## Supplementary Material

Supplementary Information Supplementary Figures 1-9, Supplementary Tables 1-5 and Supplementary References

Supplementary Data 1 List of experimentally observed PTMs within primary degrons and degron flanking residues annotated in UniProt.

Supplementary Data 2 List of residues flanking primary degrons that undergo post-translational modifications with experimental annotation about the effect of mutations.

Supplementary Data 3 Lists of lysine residues for the four datasets used (Deg, Others, Ubsites and Non-Ubsites).

Supplementary Data 4 Lists of proteins with characterized isoforms, variants and mutants that affect primary degrons.

Supplementary Data 5 Lists of proteins with characterized isoforms, variants and mutants that affect secondary degrons.

Supplementary Data 6 Lists of proteins with characterized isoforms, variants and mutants that affect tertiary degrons.

## Figures and Tables

**Table 1:** Primary degrons collected from the literature and the ELM database^4^.

E3 ligase/degron*	**Number of known instances**	**Motif pattern**†‡
APC/C (DBOX)	8	.R..L..[LIVM].
APC/C (KEN)	15	.KEN.
APC/C (ABBA)	1	[FIVL].[ILMVP][FHY].[DE].{0,3}[DEST]
APCC_TPR_1	16	.[ILM]R$
CBL (PTK)	3	[DN].**Y**[ST]..P
CBL (MET)	5	D**Y**R
COP1	7	[DE][DE].{2,3}VP[DE]
CRL4_CDT2_1	6	[NQ]{0,1}..[ILMV][ST][DEN][FY][FY].{2,3}[KR]{2,3}[^DE]
CRL4_CDT2_2	1	[NQ]{0,1}..[ILMV]T[DEN][HMFY][FMY].{2,3}[KR]{2,3}[^DE]
Kelch_KEAP1_1	13	[DNS].[DES][TNS]GE
Kelch_KEAP1_2	1	QD.DLGV
Kelch_actinfilin	1	[AP]P[MV][IM]V
Kelch_KLHL3	4	E.EE.E[AV]DQH
MDM2_SWIB	5	F[^P]{3}W[^P]{2,3}[VIL]
Nend_Nbox_1	1	^M{0,1}[FYLIW][^P]
Nend_UBRbox_1	2	^M{0,1}[RK][^P].
Nend_UBRbox_2	1	^M{0,1}([ED]).
Nend_UBRbox_3	1	^M{0,1}([NQ]).
Nend_UBRbox_4	8	^M{0,1}(C).
ODPH_VHL_1	8	[IL]A(**P**).{6,8}[FLIVM].[FLIVM]
SCF_COI1_1	6	..[RK][RK].SL..F[FLM].[RK]R[HRK].[RK].
SCF_FBW7_1	7	[LIVMP].{0,2}(**T**)P..([**ST**])
SCF_FBW7_2	2	[LIVMP].{0,2}(**T**)P..E
SCF_SKP2-CKS1_1	3	..[DE].(**T**)P.K
SCF_TIR1_1	7	.[VLIA][VLI]GWPP[VLI]...R.
SCF-TRCP1	18	D(**S**)G.{2,3}([**ST**])
SIAH	8	.P.A.V.P[^P]
SPOP	13	[AVP].[ST][ST][ST]

ELM, eukaryotic linear motif.

^*^The two CBL ligase motifs (PTK and MET) were obtained from Ng *et al*.^69^. Motif patterns for all five N-end rule degrons were present in ELM; however, four of the five degron types (Nend_Nbox_1, Nend_UBRbox_1, 2 and 3) had zero instances in ELM. Lists of experimentally validated substrates containing these motifs were therefore obtained from Varshavsky^70^. The ABBA motif recognized by the APC/C has been experimentally characterized, but is currently under curation in ELM (as a candidate motif). All the remaining degron categories (and their corresponding target substrates) have been compiled in the ELM database under the DEG (degron) motif category. Only instances that have been experimentally validated to be true positives were included. Detailed descriptions of the primary degrons are provided in Supplementary Table 1 and details about the motif instances are provided in Supplementary Table 2.

^†^The motif pattern uses the following nomenclature: ‘.' specifies any amino acid type, ‘[X]' specifies the allowed amino acid type(s) at that position, ‘^X' at the beginning of the pattern specifies that the sequence starts with amino acid type X, ‘[^X]' means that the position can have any amino acid other than type X, numbers specified as the following ‘X{*x*,*y*}', where *x* and *y* specify the minimum and maximum number of ‘X' amino acid type required at that position. ‘$' sign implies the C-terminal of the protein chain.

^‡^Conserved residue positions within the primary degron that are known to be posttranslationally modified (for example, phosphorylation and proline hydroxylation) are shown in boldface (PTM data from UniProt^61^).
