# Supplementary material for: Tripartite degrons confer diversity and specificity on regulated protein degradation in the ubiquitin-proteasome system
Source: Nat Commun. 2016 Jan 6;7:10239. doi: 10.1038/ncomms10239 (PMC4729826; doi:10.1038/ncomms10239)
Supplement: Supplementary Information — Supplementary Figures 1-9, Supplementary Tables 1-5 and Supplementary References [file ncomms10239-s1.pdf]

Supplementary Figure 1. The complexity of cellular regulatory systems responsible for transcription ('regulome'), protein function ('motifome') and degradation ('degrome') by representative numbers of entities (proteins and regulatory elements) involved. Regulome: large-scale interplay between transcription factors (~1700–1900 [1]) and DNA elements within the human genome [2] regulate gene transcription (see also main text). Motifome: a large number of PTM types, PTM enzymes and regulatory peptide motifs ensure proper protein function. The numbers of kinases [3], phosphosites [4] and representative motif elements [5] within the human proteome are shown (details in the main text). Degrome: numbers of human ubiquitin E3 ligases, number of characterized primary degron types (Table 1 and Supplementary Table 1) and their experimentally validated instances (Supplementary Table 2). The details of the experimentally validated human substrates (i.e., 93) are provided in Supplementary Table 2. 6024 human substrates were predicted to carry one or more of the 25 primary degrons that have been observed to also occur in human/mammalian proteins. SLiMSearch3 software (http://bioware.ucd.ie/~compass/biowareweb/Server_pages/slimsearch3.php) was used for predictions, and then putatively functional, degron-containing sequences were filtered (on the basis of evolutionary conservation and disorder profiles; these parameters have been validated as being clear indicators for enriching functional motifs [6]) to obtain high-confidence hits. Thus, assuming ~20000-22000 human proteins, the fraction of the proteome that carries high-confidence predicted hits to the 25 characterized motif types is on the order of 30% (shown on the pie-chart above the degradome bars).

(Figure on the following page)

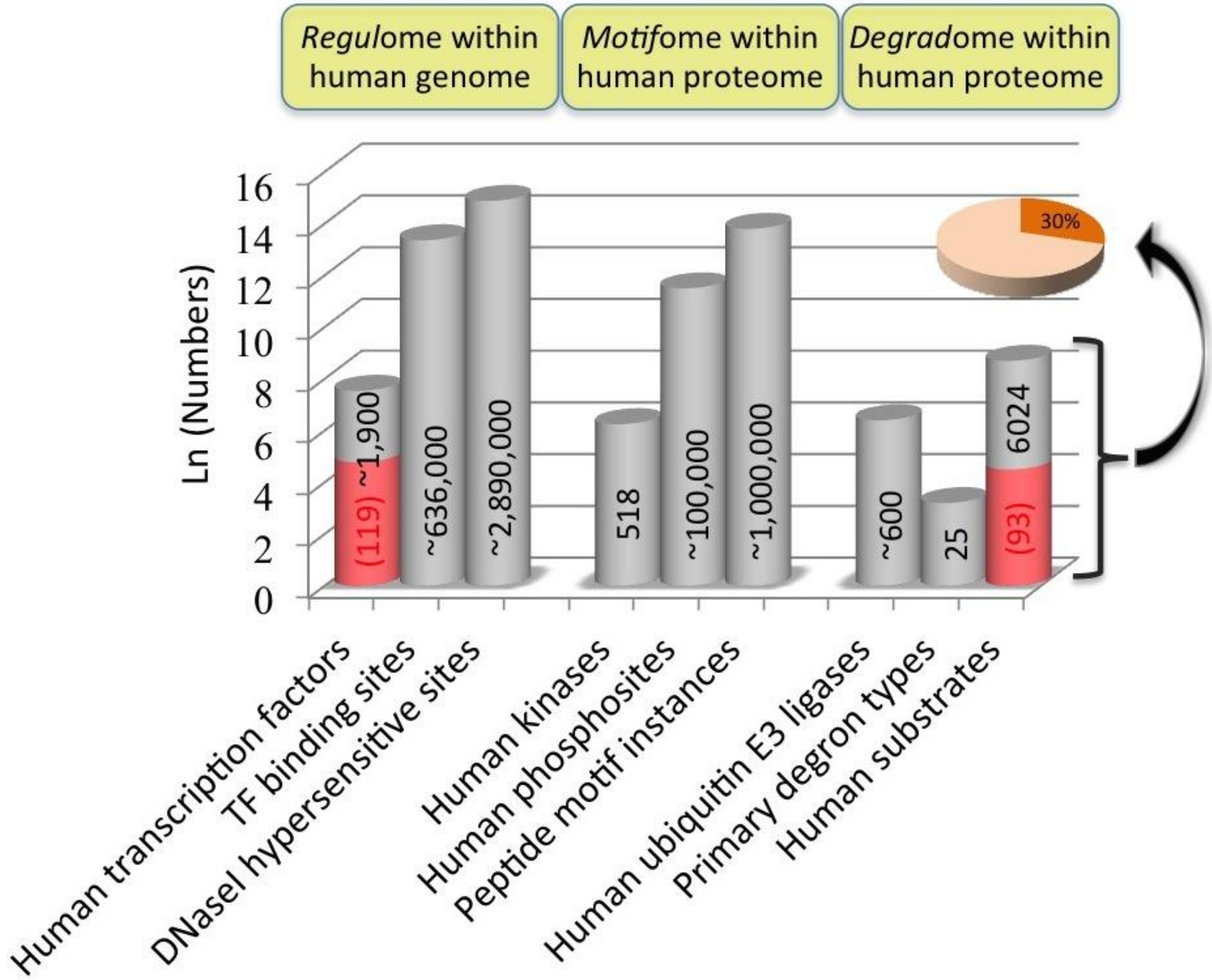
Regulome within human genome
Motifome within human proteome
Degradome within human proteome
Ln (Numbers)
16
14
12
10
8
6
4
2
0
(119) ~1,900
~636,000
~2,890,000
518
~100,000
~1,000,000
~600
25
(93)
6024
30%
Human transcription factors
TF binding sites
DNaseI hypersensitive sites
Human kinases
Human phosphosites
Peptide motif instances
Human ubiquitin E3 ligases
Primary degron types
Human substrates

Supplementary Figure 2. DynaMine S2 scores (protein backbone N-H S2 order parameters) [7] that serve as an estimate of the local backbone dynamics. Primary degron sequences (red), their flanking residues (10 neighboring residues, in both N- and C-terminal directions; in blue), and the remainder of the protein sequences (grey). 171 primary degron instances (names and UniProt IDs are given in Supplementary Table 2) were used as input. S2 scores below 0.7 indicate high flexibility, 0.7-0.8 indicates context-dependent flexibility and values >0.8 indicate structured/rigid backbone regions.

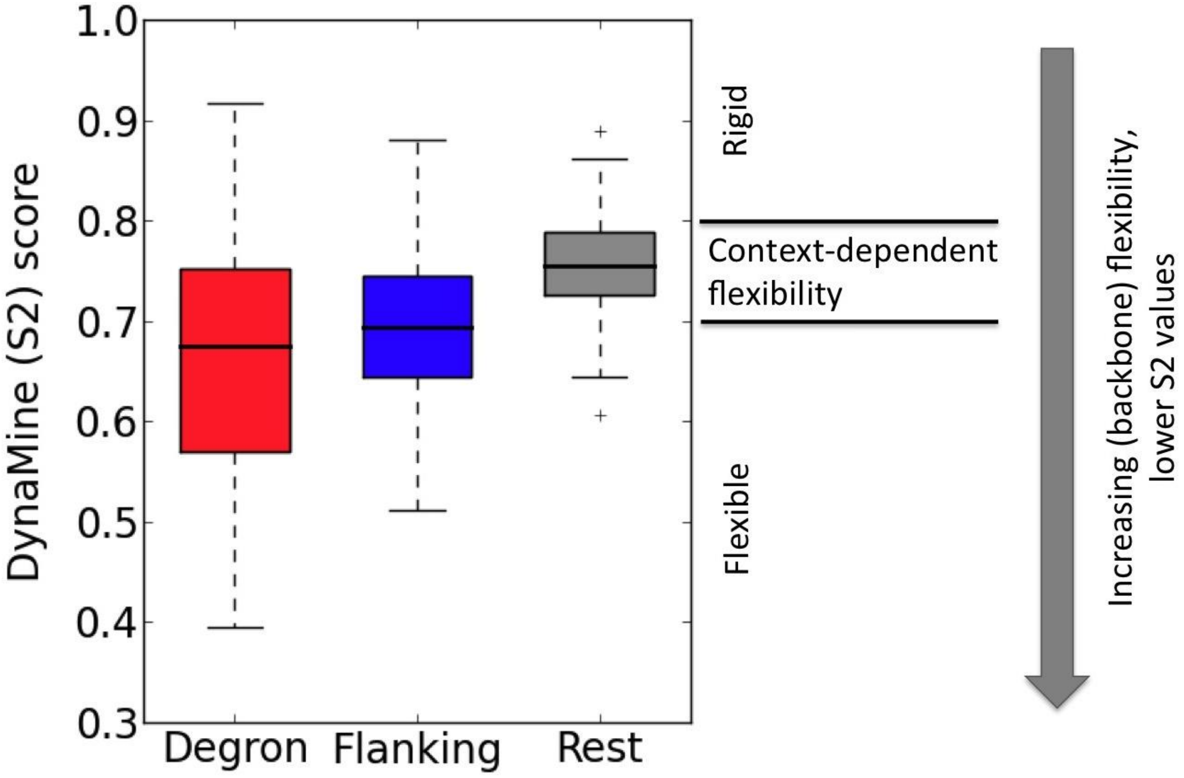

Supplementary Figure 3. Pie chart showing the observed SSE distribution of primary degron residues in the bound state (data from 23 PDB structures of degron-E3 ligase complexes, see Supplementary Table 4). Molecular figures are shown for four representative examples from different SSE types that the degron sequences form in complex with the E3 ligase. E3 ligase/E3 adaptor subunit is shown as grey surface and the substrate primary degrons are shown as cartoon (red). PDB codes for the four structures are also indicated.

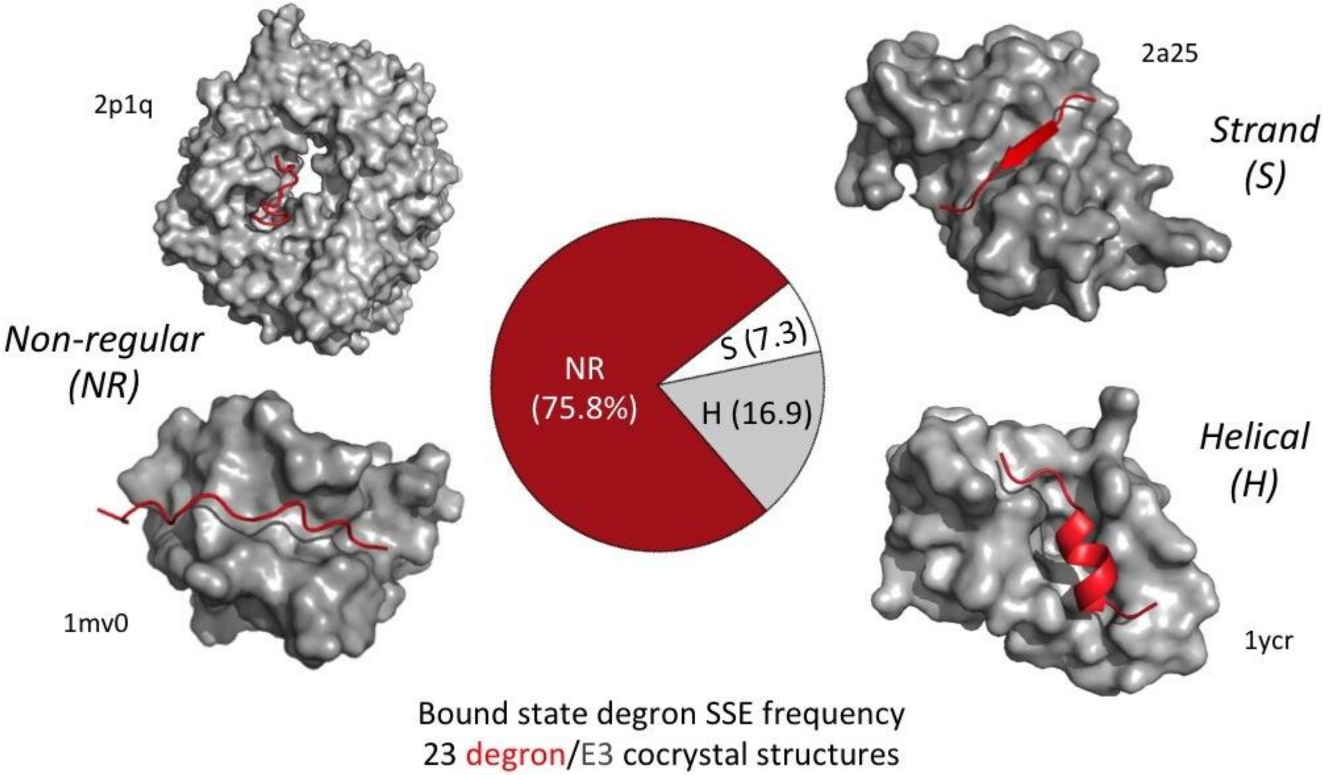

Supplementary Figure 4. Histograms showing the SPINE-X accessible surface area (ASA) values calculated for all 157 proteins of the primary degron dataset, segregated by amino acid type. Along the x-axis, ASA values (in square Angstroms) are divided into 10Å^2 bins. Along the y-axis, the bar height corresponds to the number of residues of the given amino acid type with an ASA corresponding to each bin. The total number of residues for each amino acid type is shown below the amino acid name. Blue vertical lines represent the average SPINE-X predicted ASAs for each amino acid type, whereas the red vertical lines represent the average observed ASAs obtained from a non-redundant set of PDB structures [8].

(Figure on the following page)

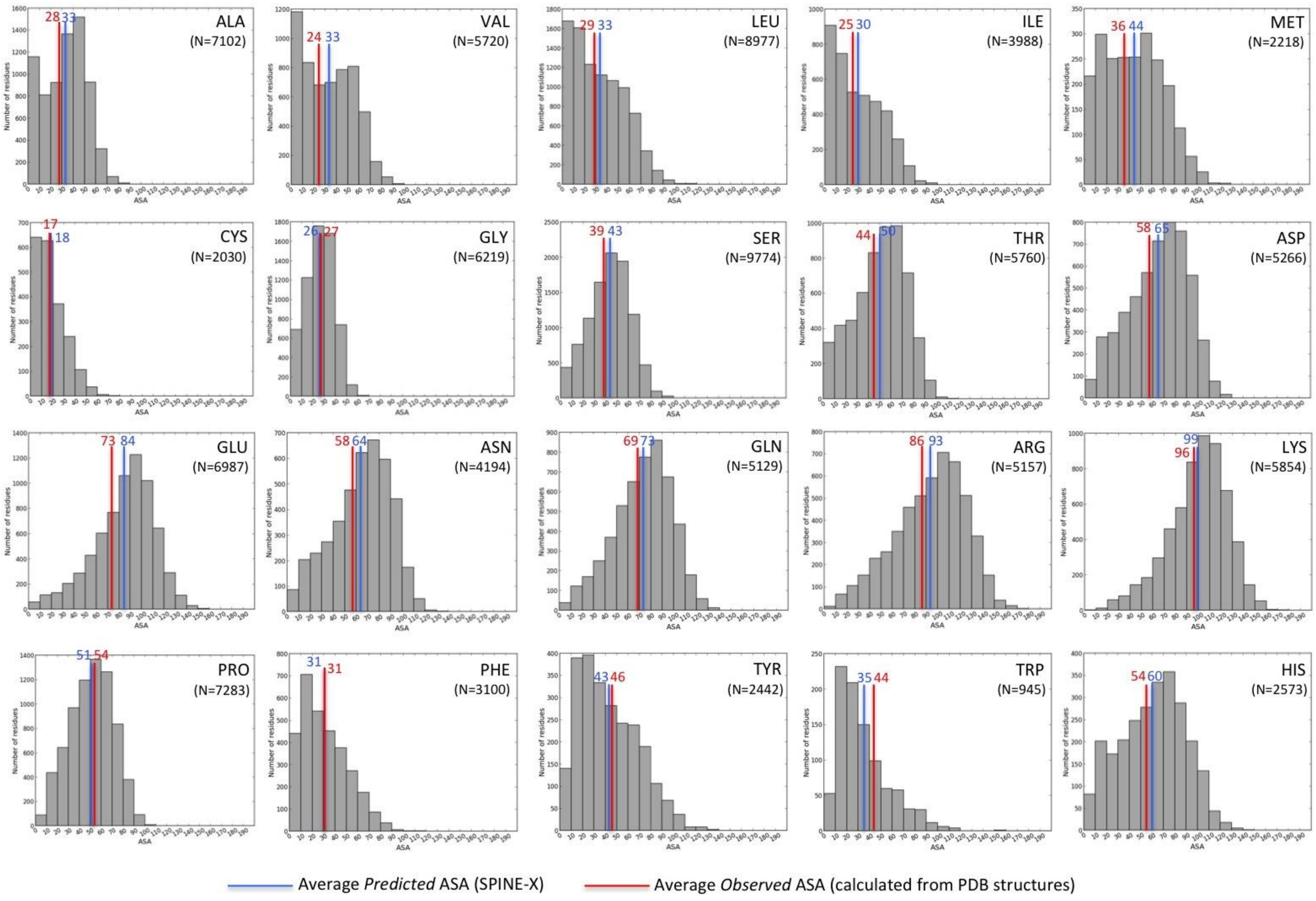
ALA (N=7102) 28 33
VAL (N=5720) 24 33
LEU (N=8977) 29 33
ILE (N=3988) 25 30
MET (N=2218) 36 44
CYS (N=2030) 17 18
GLY (N=6219) 26 27
SER (N=9774) 39 43
THR (N=5760) 44 50
ASP (N=5266) 58 65
GLU (N=6987) 73 84
ASN (N=4194) 58 64
GLN (N=5129) 69 73
ARG (N=5157) 86 93
LYS (N=5854) 99 96
PRO (N=7283) 51 54
PHE (N=3100) 31 31
TYR (N=2442) 43 46
TRP (N=945) 35 44
HIS (N=2573) 54 60
Number of residues
ASA
Average *Predicted* ASA (SPINE-X)
Average *Observed* ASA (calculated from PDB structures)

Supplementary Figure 5. Average Z-ASA values for the primary degron and flanking regions are shown relative to the (background) ASA Zscore distribution for the entire dataset. This enables a relative comparison of the average surface accessibility of degron and degron flanking regions, relative to the background ASA distribution calculated for the whole protein dataset (shown separately for the 28 primary degron types). First, SPINE-X ASA predictions for all residues of the 157 proteins of the primary degron dataset were made. Absolute ASAs were converted into Z-scores using residue-specific ASA distributions (shown in Supplementary Figure 4). This overall Z-score distribution for all residue types is shown as grey bars in each of the sub-figures. Higher Z-scores indicate higher relative accessibility. **Dotted vertical lines** show the location of the **average Z-ASA** of the primary degron residues (red) and average Z-ASA of its flanking regions (blue), considering all the degron instances belonging to each class.

(Figures on the following pages 8-11)

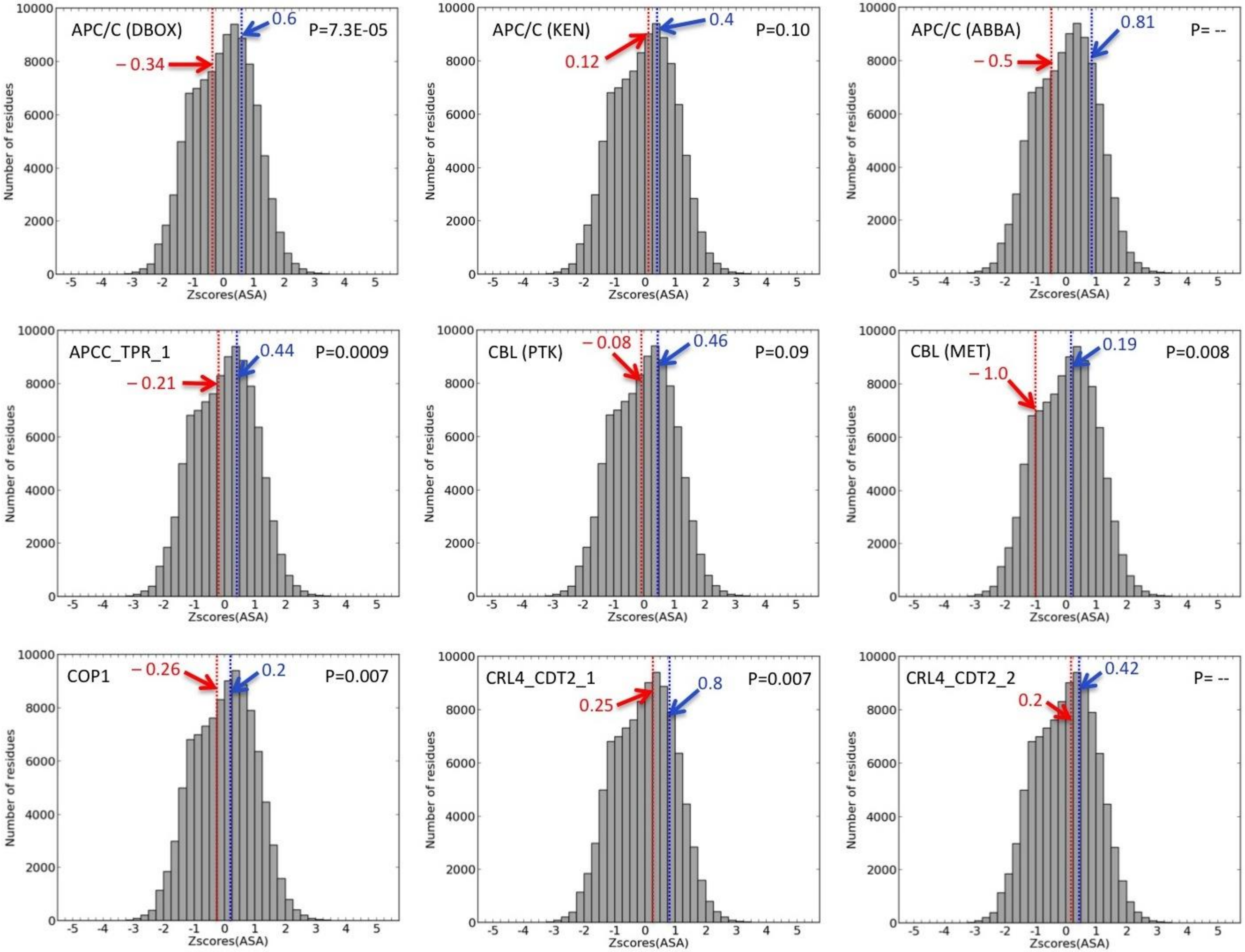
APC/C (DBOX)
P=7.3E-05
− 0.34
0.6
APC/C (KEN)
P=0.10
0.12
0.4
APC/C (ABBA)
P= --
− 0.5
0.81
APCC_TPR_1
P=0.0009
− 0.21
0.44
CBL (PTK)
P=0.09
− 0.08
0.46
CBL (MET)
P=0.008
− 1.0
0.19
COP1
P=0.007
− 0.26
0.2
CRL4_CDT2_1
P=0.007
0.25
0.8
CRL4_CDT2_2
P= --
0.2
0.42
Number of residues
Zscores(ASA)

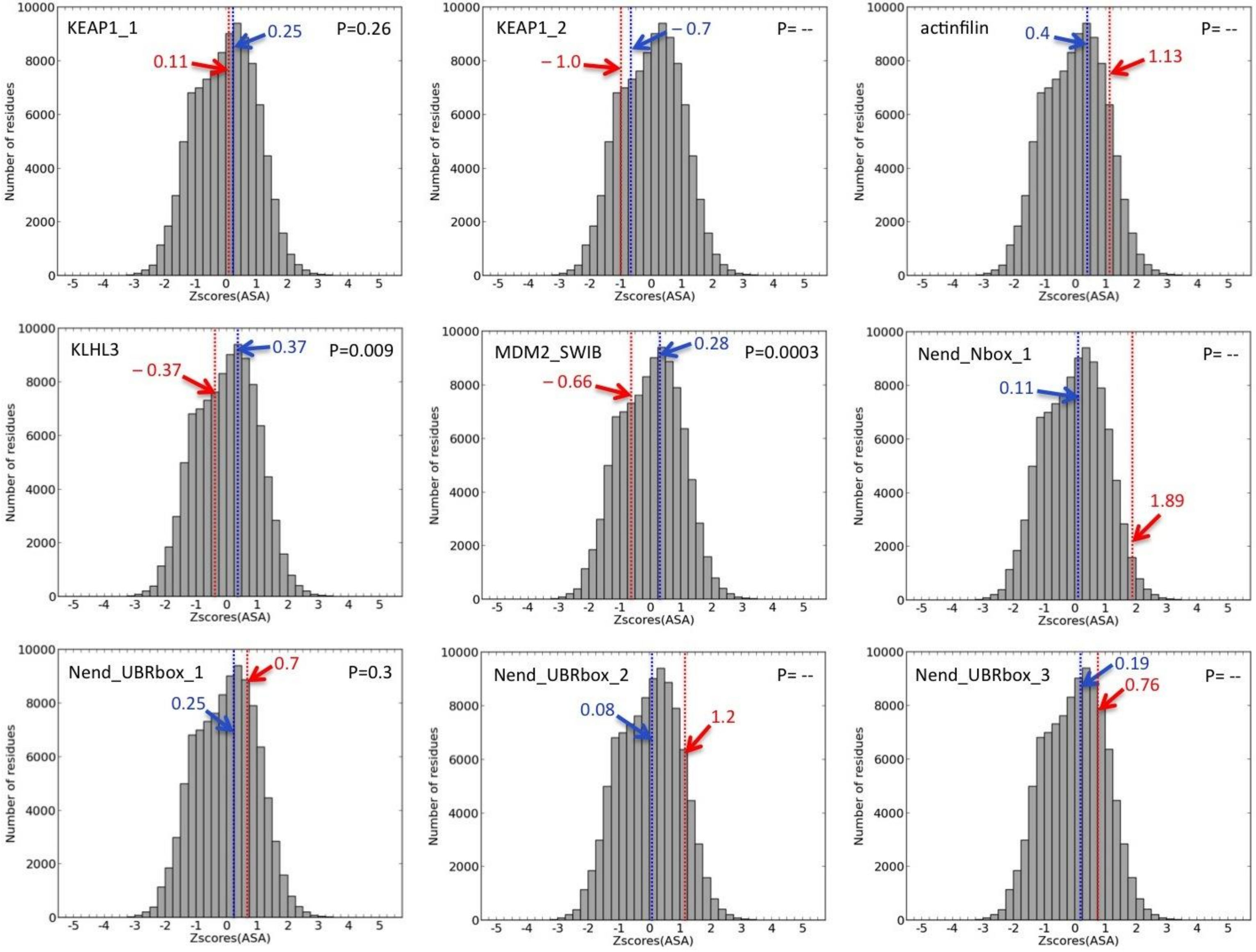
KEAP1_1
P=0.26
0.25
0.11
KEAP1_2
P= --
– 0.7
– 1.0
actinfilin
P= --
0.4
1.13
KLHL3
P=0.009
0.37
– 0.37
MDM2_SWIB
P=0.0003
0.28
– 0.66
Nend_Nbox_1
P= --
0.11
1.89
Nend_UBRbox_1
P=0.3
0.7
0.25
Nend_UBRbox_2
P= --
0.08
1.2
Nend_UBRbox_3
P= --
0.19
0.76
Number of residues
Zscores(ASA)

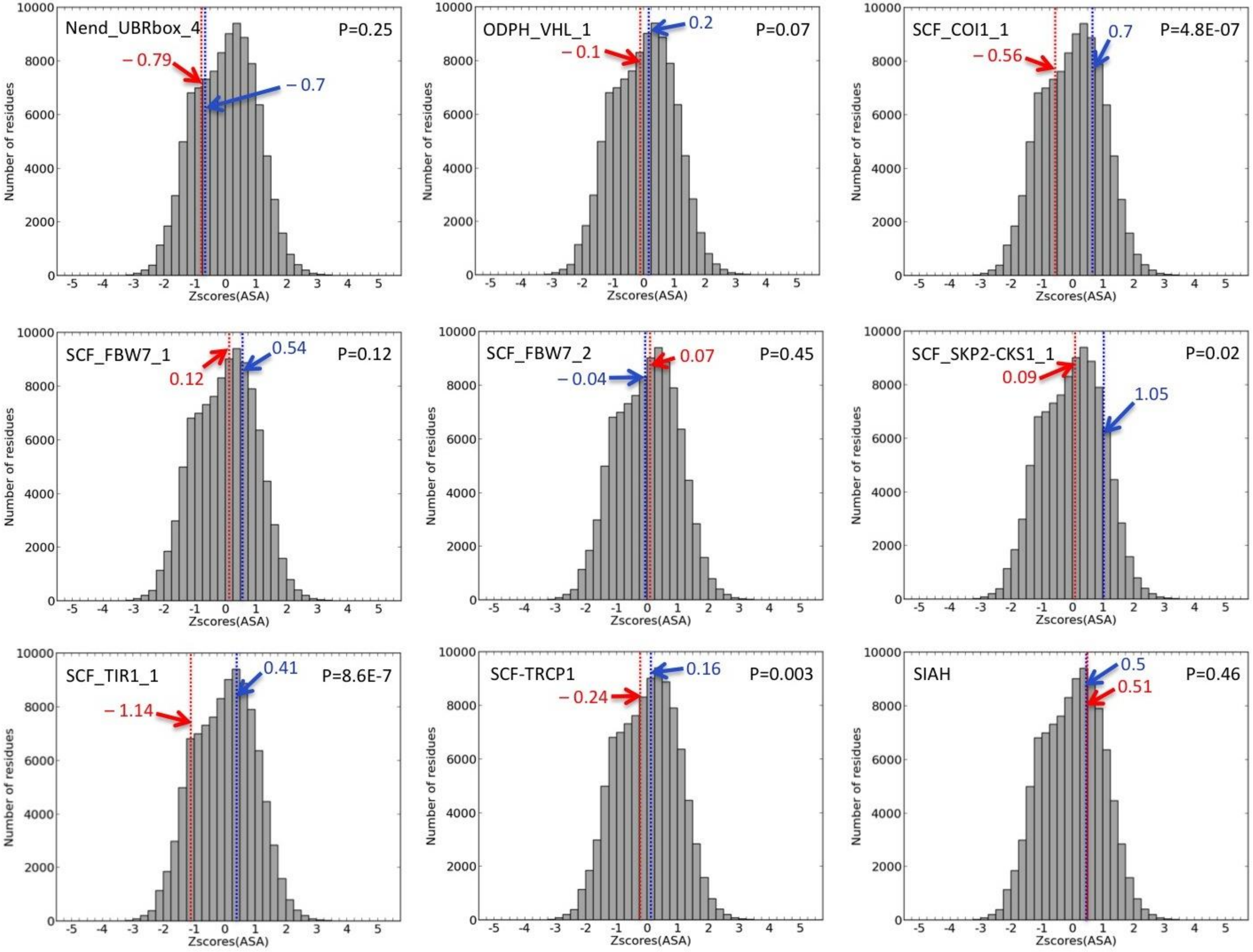

Nend_UBRbox_4
P=0.25
− 0.79
− 0.7
ODPH_VHL_1
P=0.07
− 0.1
0.2
SCF_COI1_1
P=4.8E-07
− 0.56
0.7
SCF_FBW7_1
P=0.12
0.12
0.54
SCF_FBW7_2
P=0.45
− 0.04
0.07
SCF_SKP2-CKS1_1
P=0.02
0.09
1.05
SCF_TIR1_1
P=8.6E-7
− 1.14
0.41
SCF-TRCP1
P=0.003
− 0.24
0.16
SIAH
P=0.46
0.5
0.51
Number of residues
Zscores(ASA)

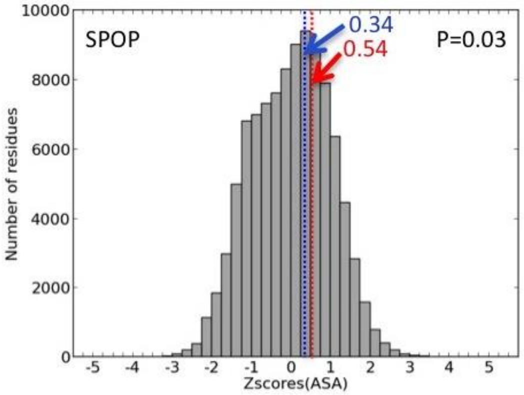
SPOP
P=0.03
0.34
0.54
Number of residues
Zscores(ASA)
10000
8000
6000
4000
2000
0
-5
-4
-3
-2
-1
0
1
2
3
4
5

Supplementary Figure 6. Evolutionary conservation of Ser, Thr and Tyr [STY] residues estimated using Sequence Entropy (S) values calculated from multiple sequence alignments of orthologs (see Methods). The lower the value of 'S' for a given alignment position, the higher is the conservation of that residue. (A) Comparison of 'S' values for [STY] residues located in flanking regions (10 neighboring residues, in both N- and C-terminal directions) of primary degron segments (red color) versus all [STY] residues in proteins present in the primary degron dataset (blue color). (B) Average Sequence Entropy (<S>) values calculated for all [STY] residues in a protein (along the x-axis) versus the <S> for [STY] residues flanking the primary degron for that protein (y-axis). Each point corresponds to a protein in the primary degron dataset. Points below the diagonal indicate that the degron-flanking [STY] residues are better conserved as compared to the set of all [STY] for that protein.

(Figure on the following page)

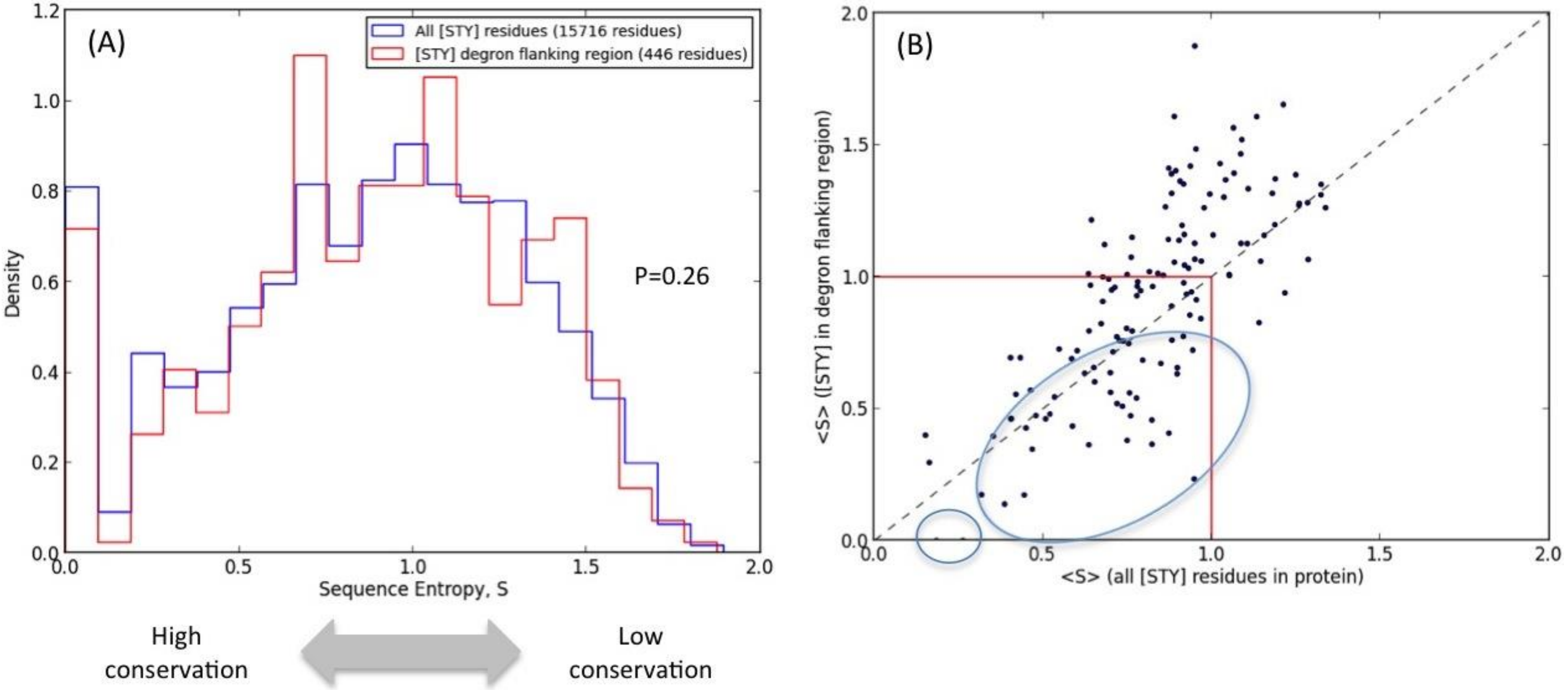
(A)
All [STY] residues (15716 residues)
[STY] degron flanking region (446 residues)
P=0.26
Density
Sequence Entropy, S
High conservation
Low conservation
(B)
<S> ([STY] in degron flanking region)
<S> (all [STY] residues in protein)

Supplementary Figure 7. Heatmap showing enrichment (green) or depletion (red) of the 20 aa types neighboring the Deg lysines (position '0') relative to the Others set (details provided in Methods and main text).

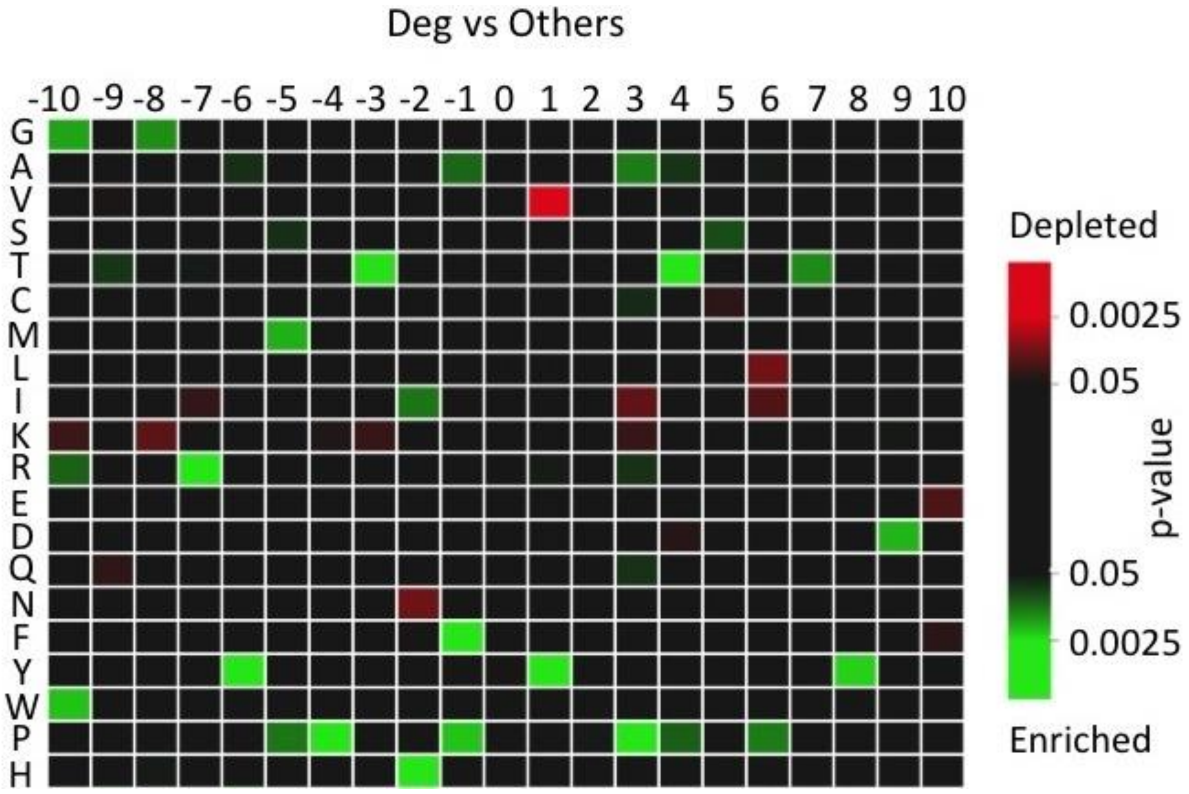

Supplementary Figure 8. Relative positioning of primary degron(s) and secondary degron(s) in specific substrates that were present in both the primary degron and Deg lysine (degradation-linked) datasets. Eleven such substrates were found and the domain organization of these proteins is shown along with the location of the primary degron and the Deg lysines (in red). Domains are colored cyan and inter-domain regions are in grey. Pfam version 27.0 was used for domain annotations. On the bottom panels, the distance (along the primary sequence) distribution of degradation-linked Deg (red) and non-degradation linked Others (blue) lysines, relative to the primary degron are shown.

(Figures on the following pages 16-27)

(A) P04637 (Cellular tumor antigen p53)

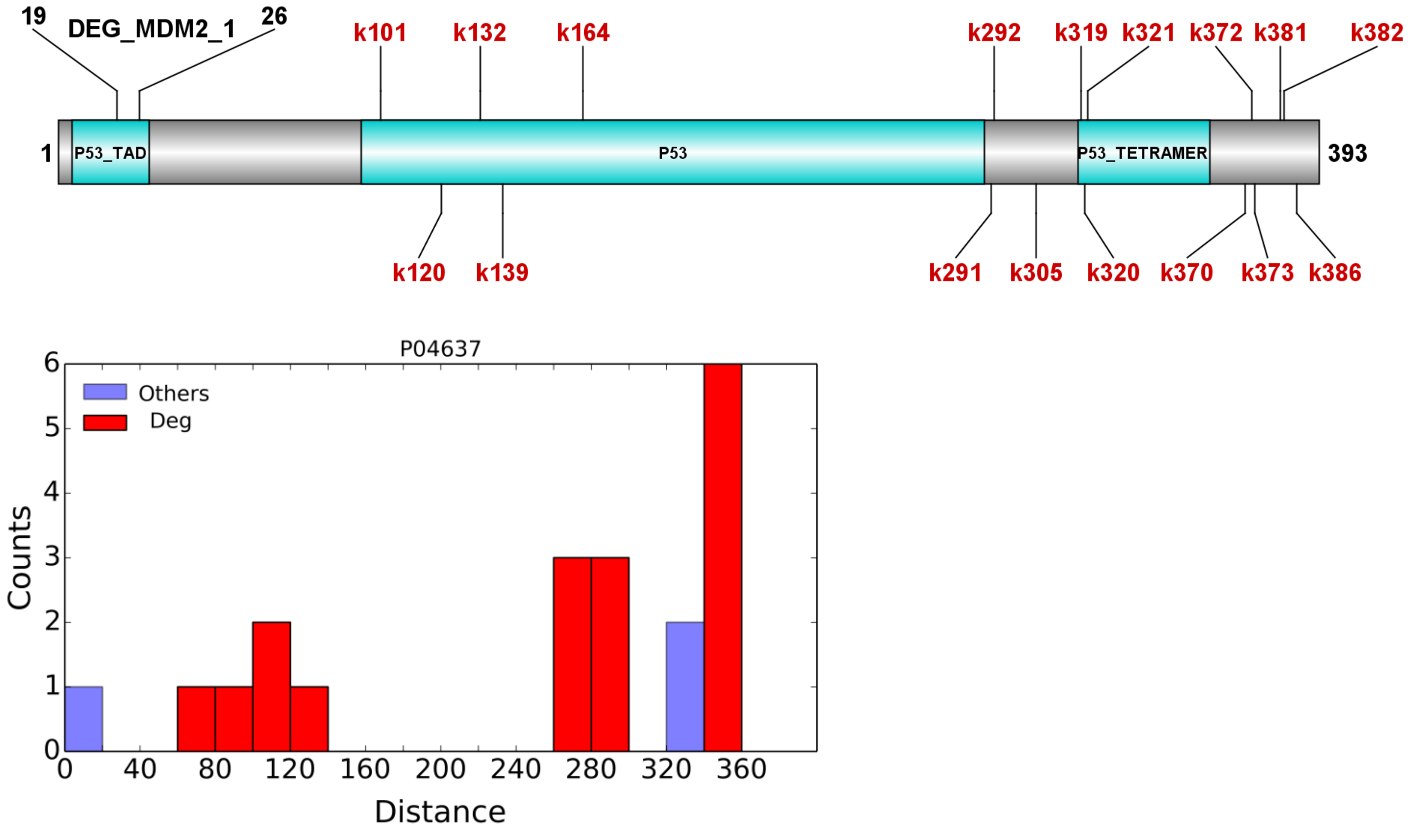

(B) P30307 (CDC25C, M-phase inducer phosphatase 3)

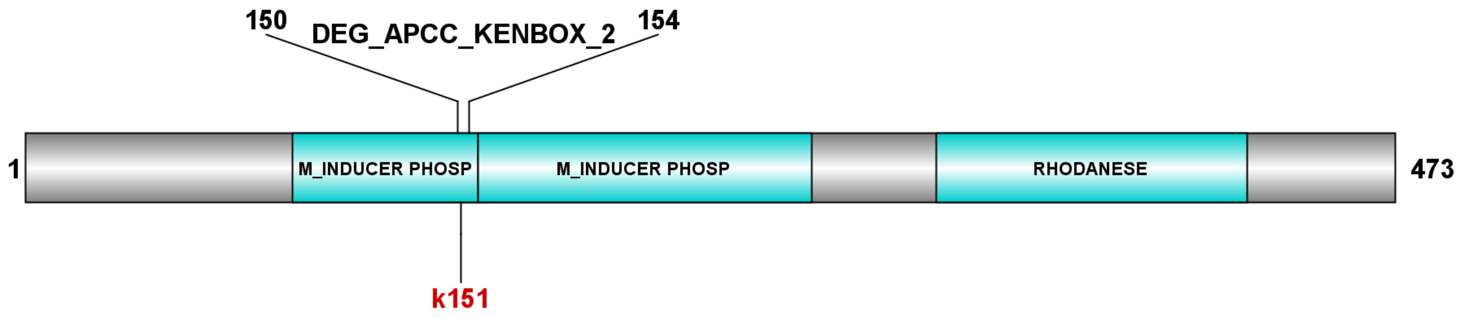

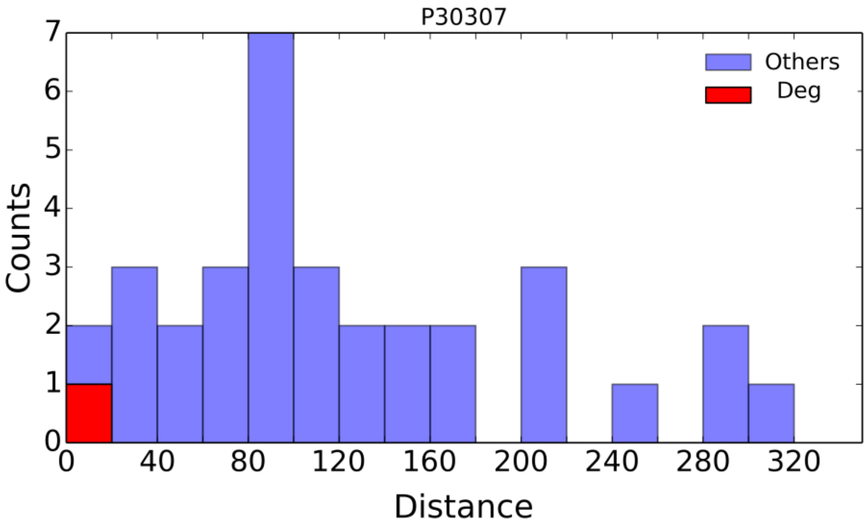

(C) P35222 (Catenin beta-1)

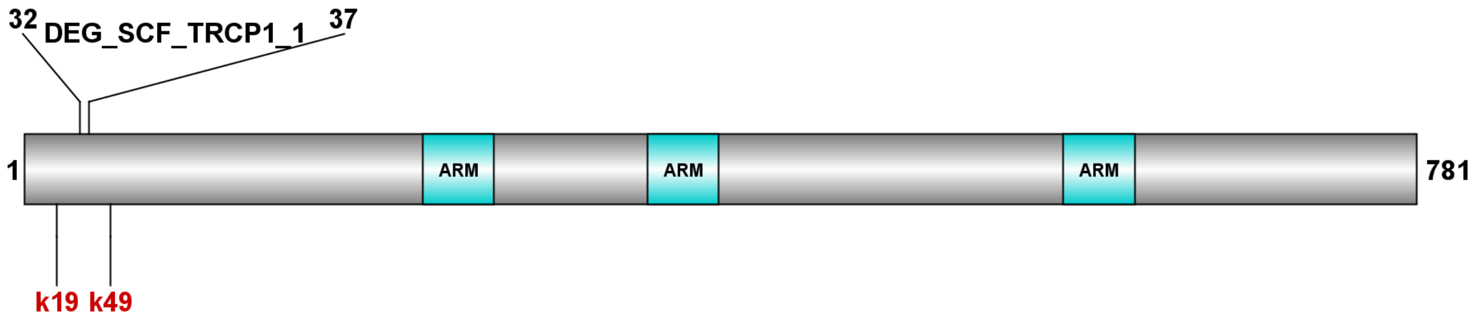

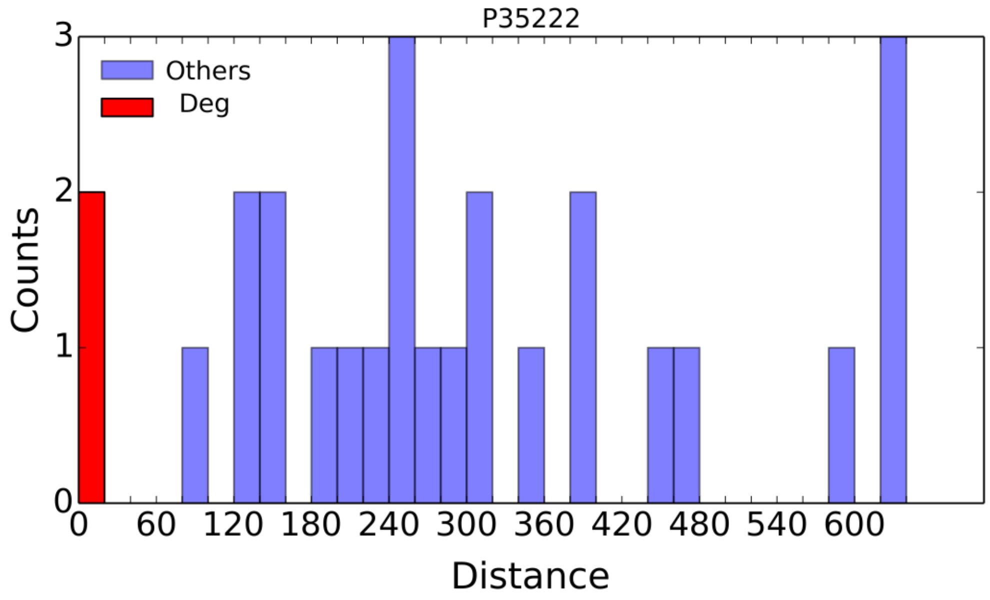

(D) P25963 (NF-kappa-B inhibitor alpha)

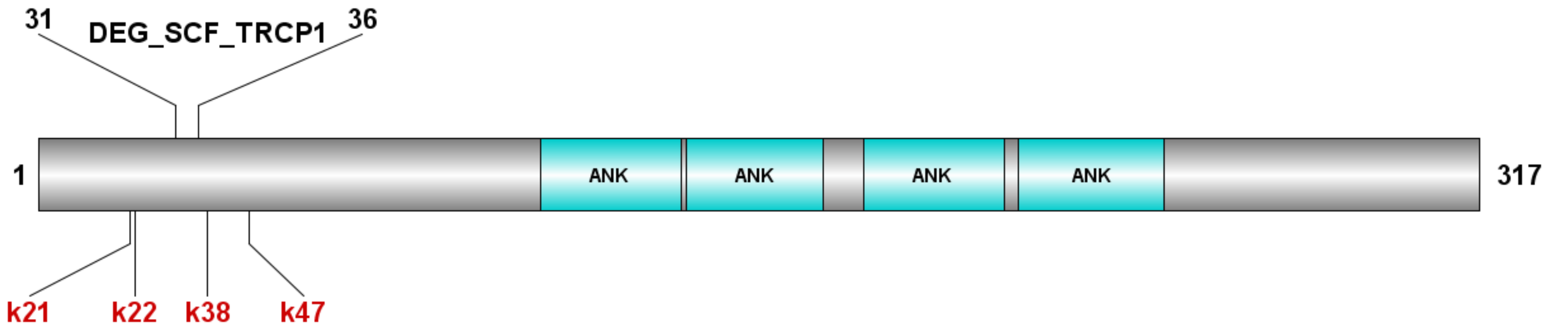

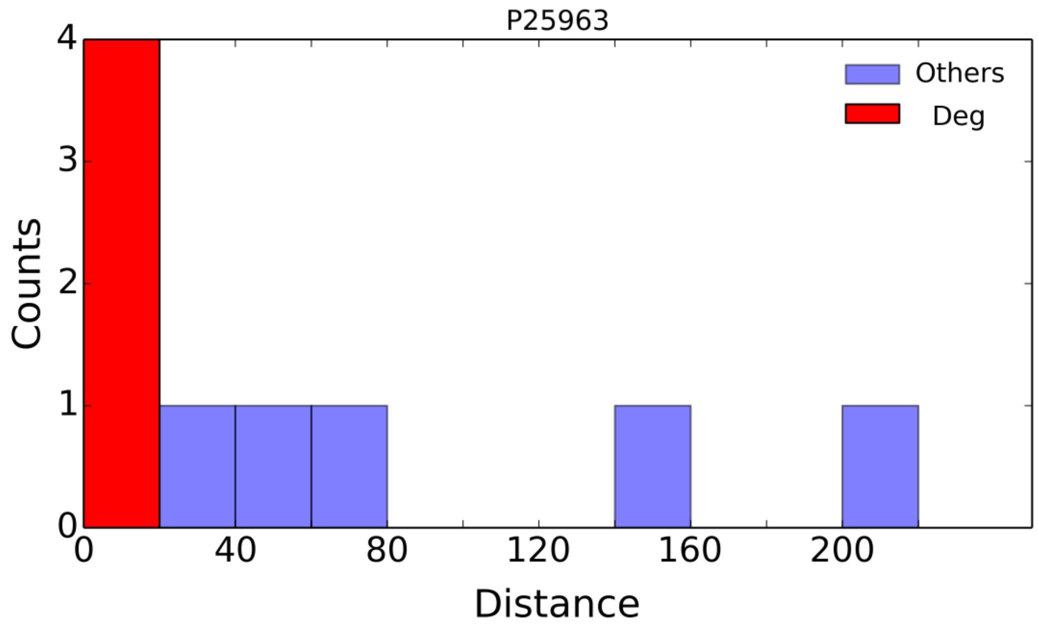

(E) P38936 (Cyclin-dependent kinase inhibitor 1)

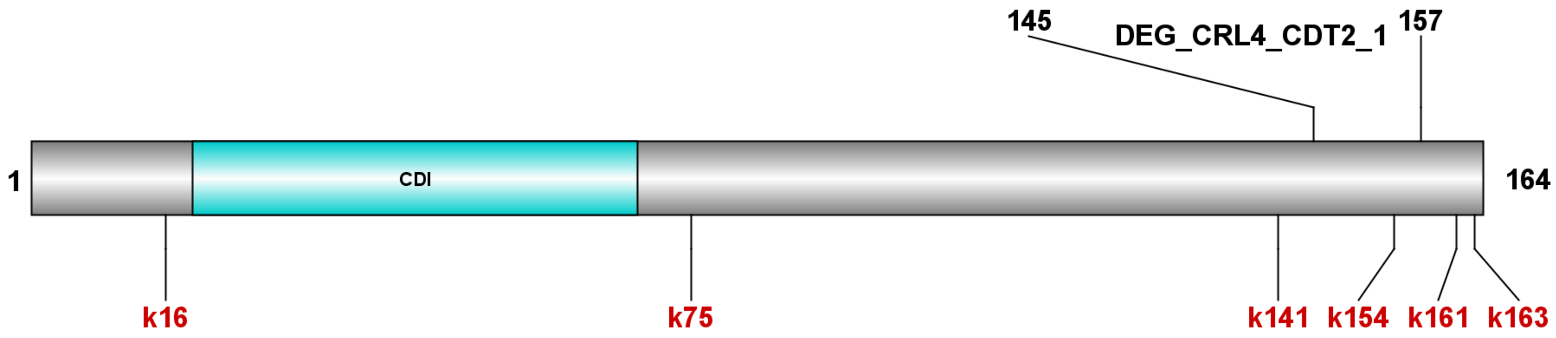

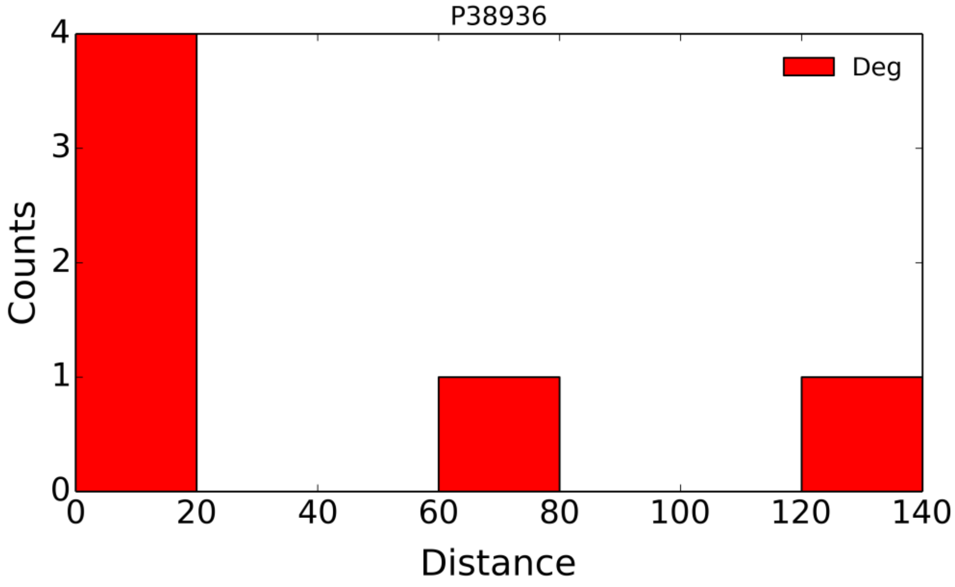

(F) P46527 (Cyclin-dependent kinase inhibitor 1B)

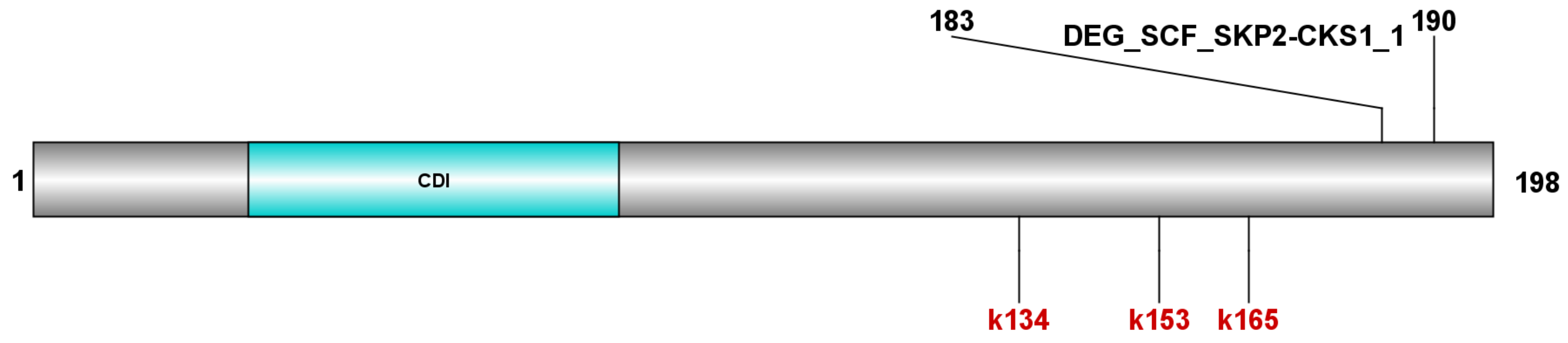

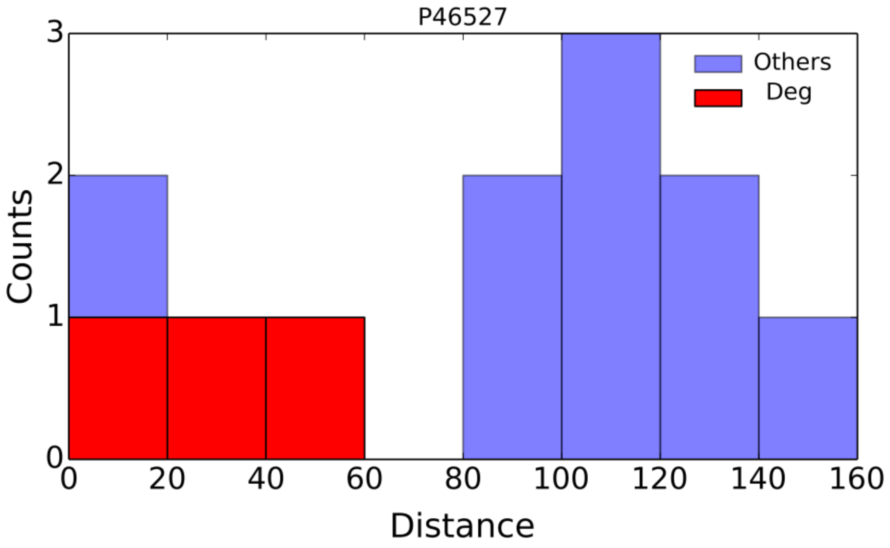

(G) Q9Y2N7 (Hypoxia-inducible factor 3-alpha)

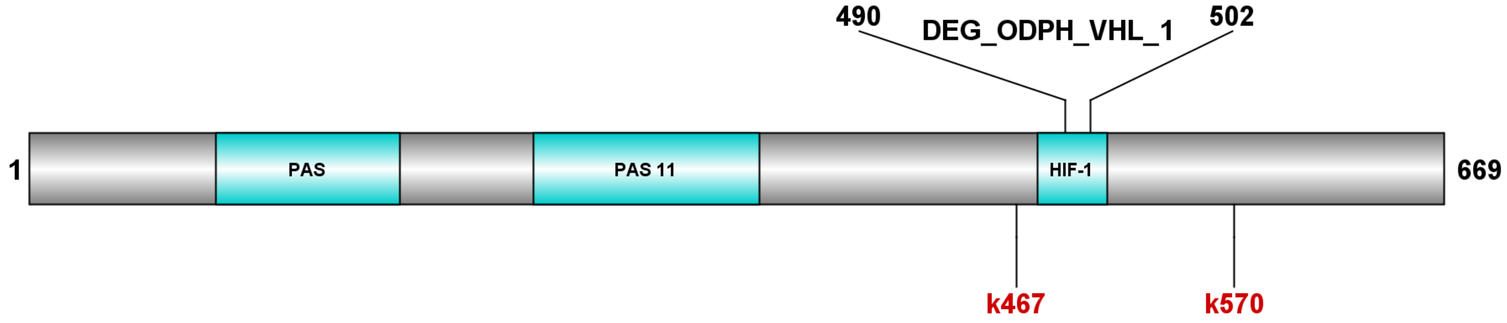

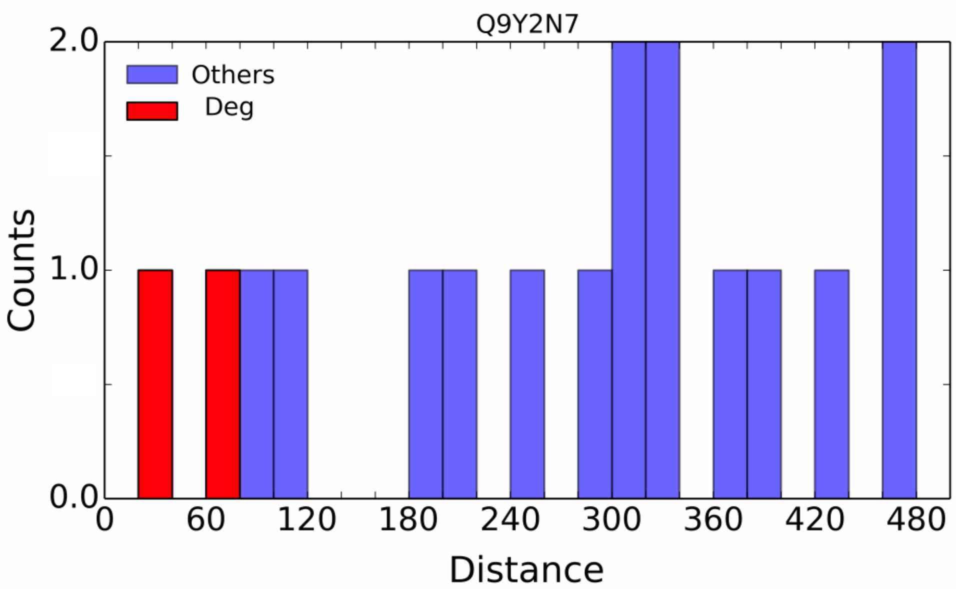

(H) Q16665 (Hypoxia-inducible factor 1-alpha)

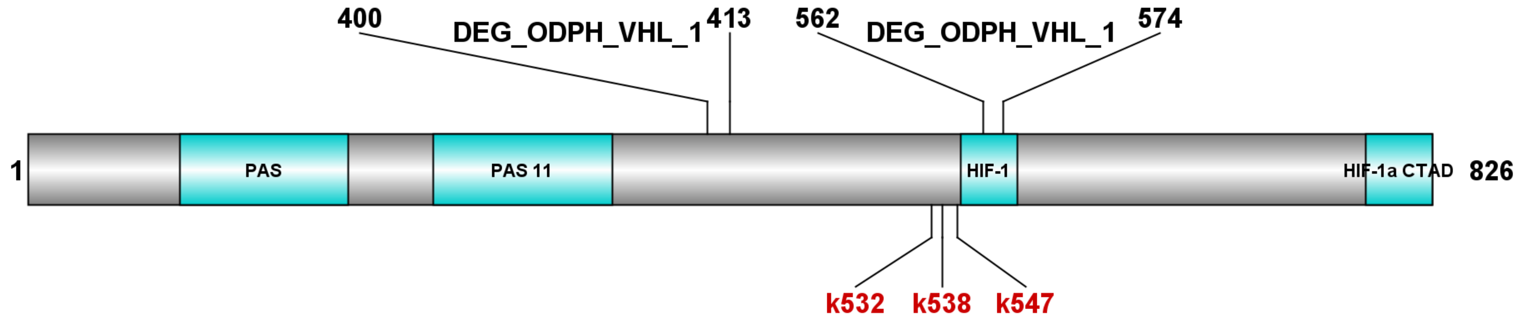

(primary degron res num 400-413)

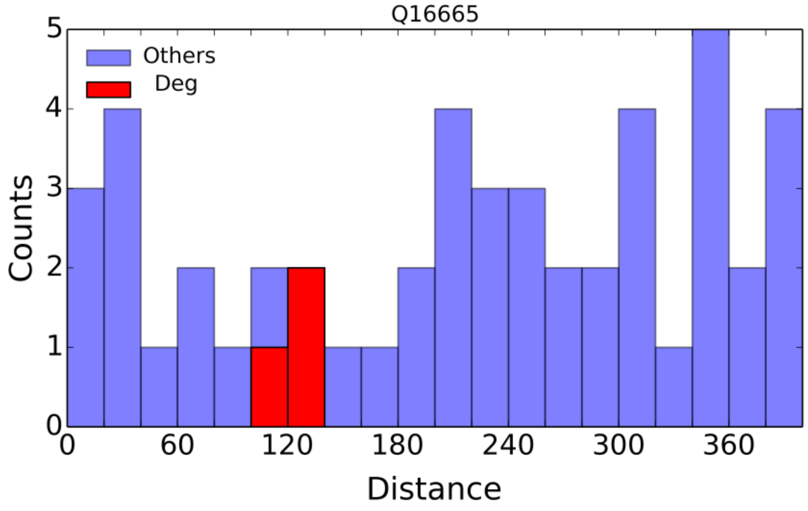

(primary degron res num 562-574)

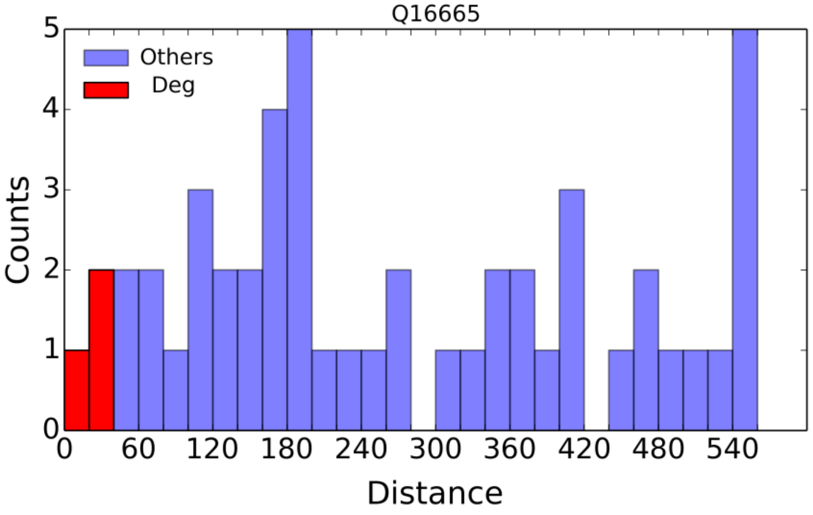

(I) Q99814 (Endothelial PAS domain-containing protein)

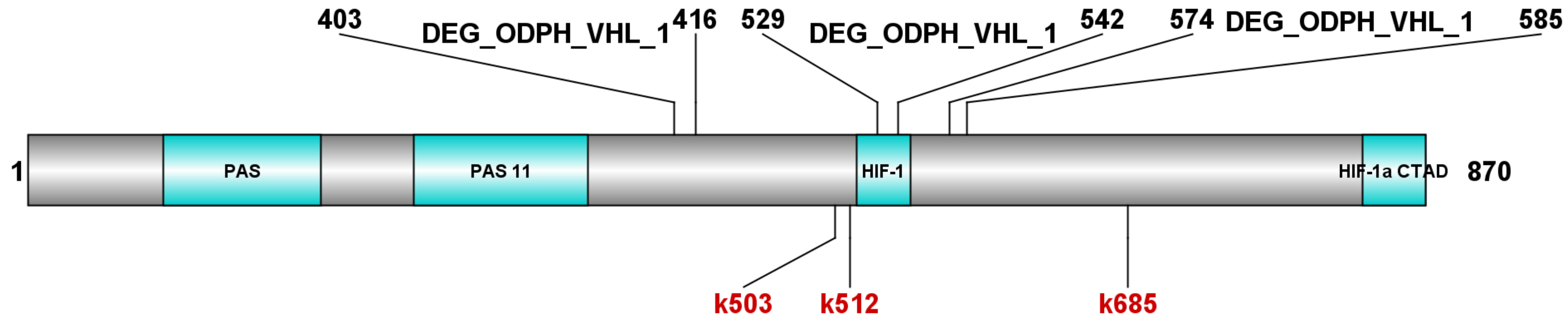

(primary degron res num 574-585)

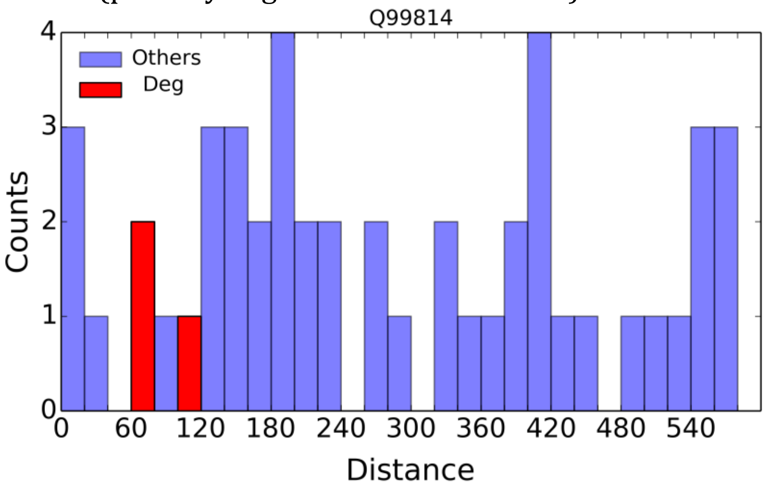

(primary degron res num 403-416)

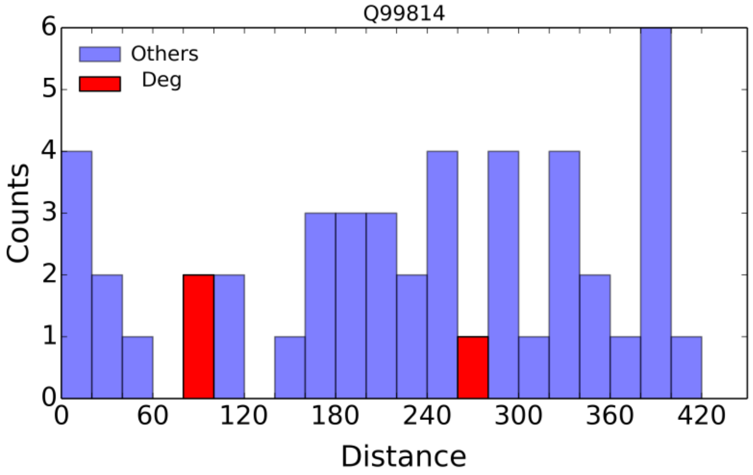

(primary degron res num 529-542)

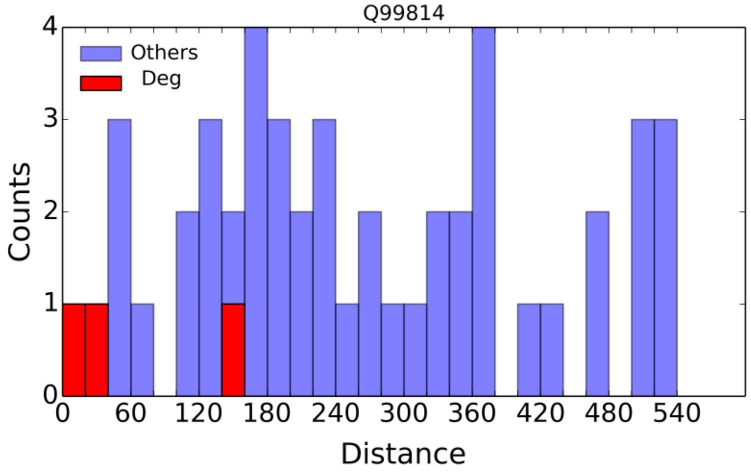

(J) P10071 (Transcriptional activator GLI3)

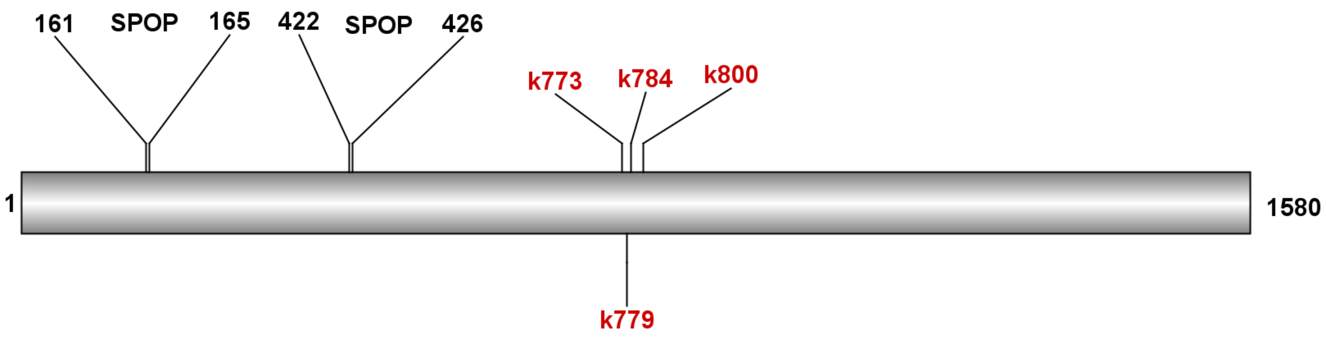

(primary degron res num 161-165)

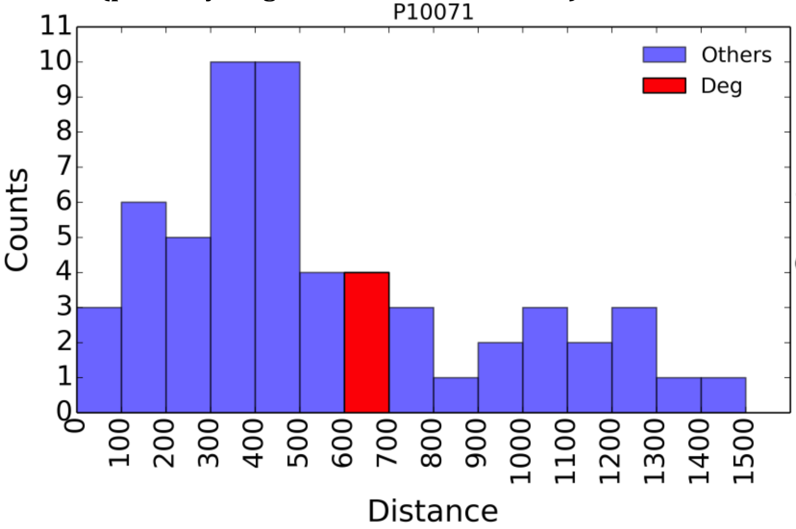

(primary degron res num 422-426)

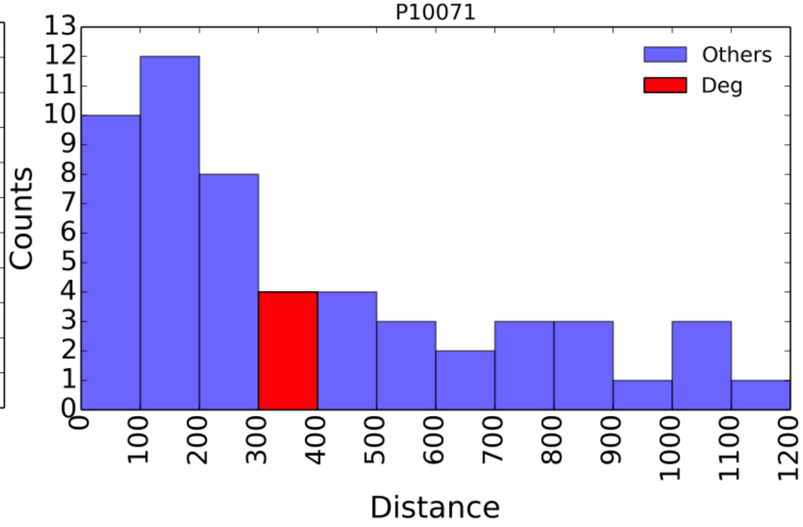

(K) P20248 (Cyclin-A2)

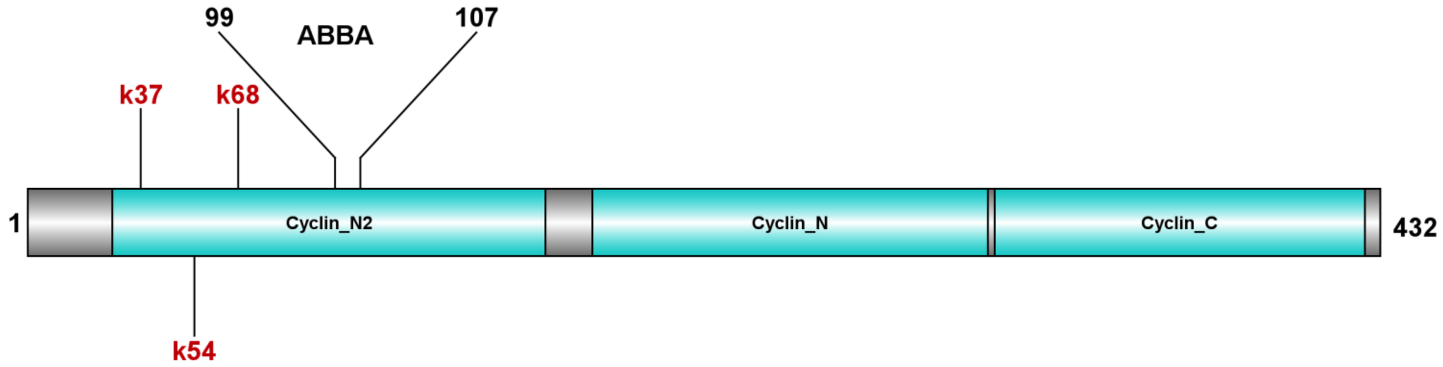

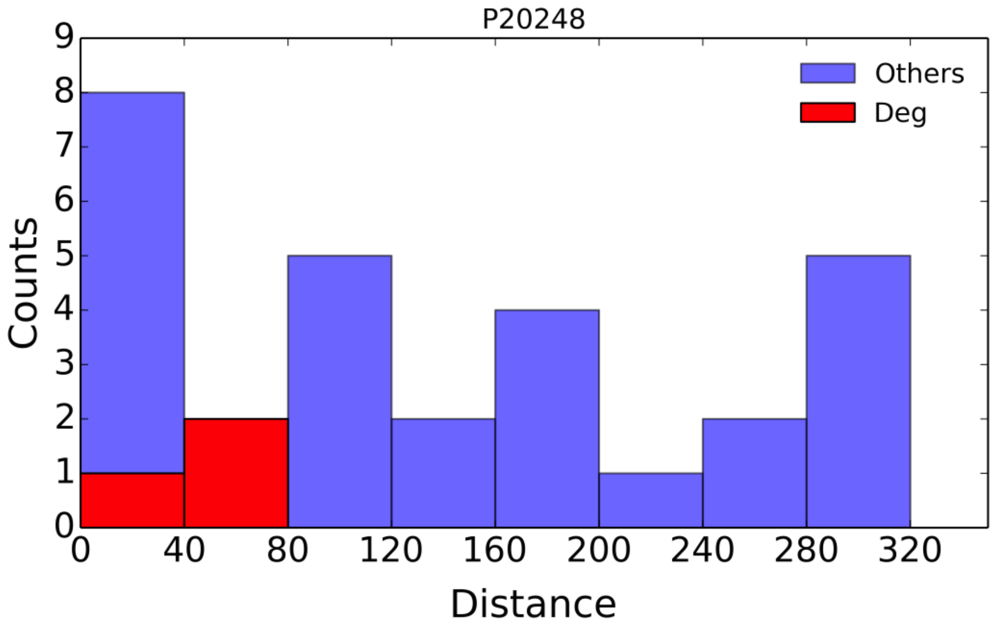

Supplementary Figure 9. Percentages of lysine residues (from each of the four lysine datasets: Deg, Others, Ubsites and Non-Ubsites) that are within a given distance (number of amino acids away) from the nearest long flexible region predicted using DynaMine. The inset shows the fraction of lysines that are located within a long flexible region. Long flexible regions are defined as sequence regions containing 20 or more consecutive residues that are predicted to be flexible (including residues that are predicted to possess context-dependent flexibility) (see Methods). Breaks of at most 3 consecutive non-flexible (rigid) residues were permitted within any long flexible region.

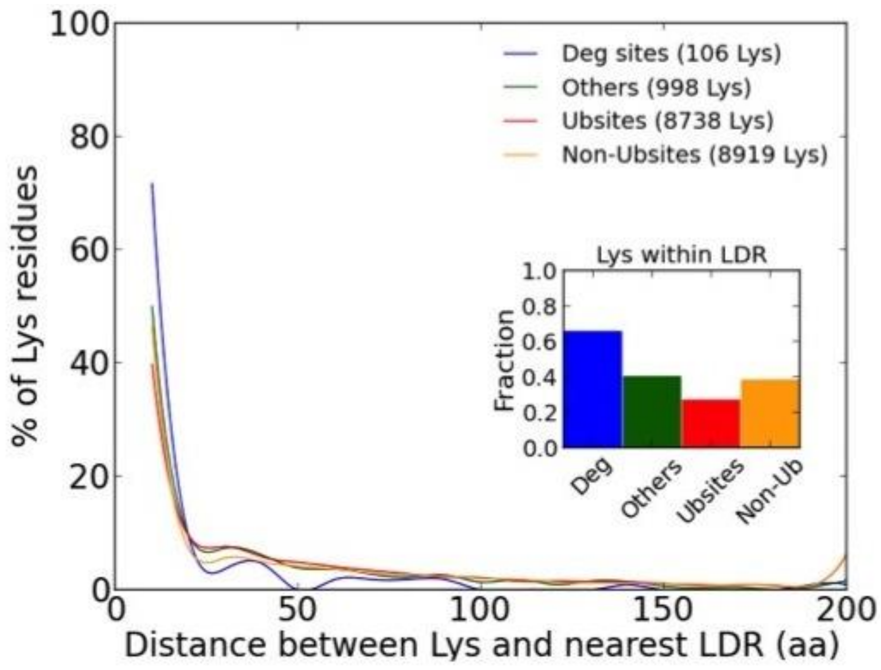

Supplementary Table 1. List of characterized primary degrons collected from the literature and the ELM database.

E3 ligase/degron	# known instances[a]	Motif pattern[b,c]	Short description[d]
APC/C (DBOX)	8	.R..L..[LIVM].	The multi-subunit E3 ligase anaphase-promoting complex/cyclosome (APC/C) uses the adaptor/coactivator proteins Cdc20 and Cdh1 (containing WD40-repeat domains) to bind to degradation substrates using the DBOX motif. DBOX containing substrates are mainly cell cycle regulatory proteins, whose regulated (and sequential) degradation is essential to the correct progress of the cell cycle [9,10].
APC/C (KEN)	15	.KEN.	This motif is also found in cell cycle proteins that are regulated by APC/C. Of the two APC adaptors, KEN-box motifs are preferentially recognized by Cdh1. The other adaptor, Cdc20, carries a KEN-motif that enables it to be degraded by APC/Cdh1 in mitotic exit and G1 [9,10].
APC/C (ABBA)	1	[FIVL].[ILMVP][FHY].[DE].{0,3}[DEST]	This motif binds to the APC/C coactivator Cdc20 and has been named 'ABBA' since it is conserved in human *A*-type cyclins, *B*UBR1, *B*UB1 and in yeast *A*cm1 [11]. However, only cyclin A is a confirmed degradation substrate that is recognized by APC/C^{Cdc20} in prometaphase and then rapidly degraded. The others bind Cdc20 and regulate APC function.
APCC_TPR_1	16	.[ILM]R$	C-terminal motif present in some APC/C substrates and in the APC/C co-activators Cdc20 and Cdh1. This motif allows binding directly to the tetratricopeptide repeat (TPR) regions present in the core subunits (mainly Apc3) of the APC/C complex [12,13].
CBL (PTK)	3	[DN].**Y**[ST]..P	Phosphorylated tyrosine motif, substrates bind to tyrosine kinase-binding (TKB) domains of CBL-family E3 ligases; involved in degradation of receptor tyrosine kinases (RTKs) and non-RTKs [14,15].
CBL (MET)	5	D**Y**R	Another consensus motif targeted by the CBL-TKB domain, found conserved among Met family proteins and plexins [14,16,17].
COP1	7	[DE][DE].{2,3}VP[DE]	The Constitutive photomorphogenesis protein (COP1) is a RING-finger E3 ligase involved both in plant (photomorphogenic development in *A. thaliana*) and mammalian (transcription regulation) protein degradation. Substrates share a negatively charged motif [18-20].
CRL4_CDT2_1	6	[NQ]{0,1}..[ILMV][ST][DEN][FY][FY].{2,3}[KR]{2,3}[^DE]	This degron is recognized by the WD40 domain of Cdc10-dependent transcript 2 (Cdt2), which acts as the substrate-binding domain in the CRL4^{Cdt2} E3 ligase complex. Found in regulatory proteins such as chromatin-associated proteins. The degron partially overlaps with a Proliferating Cell Nuclear Antigen (PCNA)-interaction motif on substrate proteins and it is believed that degron recognition is PCNA-dependent. Degradation normally occurs during S phase or following DNA damage [21].

CRL4_CDT2_2	1	[NQ]{0,1}..[ILMV]T[DEN][HMFY][FMY].{2,3}[KR]{2,3}[^DE]	A variant of the above. The two strongly conserved aromatic residue positions (in vertebrates) additionally allow substitution by Met in non-vertebrates.
Kelch_KEAP1_1	13	[DNS].[DES][TNS]GE	Keap1 is a substrate-binding adaptor protein for a Cullin 3-based multi-subunit ubiquitin ligase that binds to this acidic degron motif present in proteins involved in oxidative stress response, such as Nrf2; the degron is also found in IKKbeta and therefore also involved in the regulation of NF-kappaB signaling [22-24]. This is the primary (high-affinity) binding site and it is highly conserved from fly to human.
Kelch_KEAP1_2	1	QD.DLGV	The secondary (and weaker) KEAP1 binding motif present in degradation targets of KEAP1. This degron has been well characterized in the KEAP1 substrate Nrf2. Similar to the higher affinity degron, it also binds to the bottom of the Kelch β-propeller domain [25,26] and cooperative binding by both the degrons ensures stable interaction with KEAP1.
Kelch_actinfilin	1	[AP]P[MV][IM]V	A hydrophobic degron found in Kainate receptors such as GluR6 that is recognized by Actinfilin, an actin-binding protein that also functions as a substrate adaptor for Cullin3-based E3 Ub-ligase. The Kelch domain of Actinfilin is responsible for mediating the interaction [27].
Kelch_KLHL3	4	E.EE.E[AV]DQH	Highly acidic degron found in vertebrate WNK kinases that is recognized by Cullin3-based KLHL2 and KLHL3 E3 ligases [28]. The degron-binding Kelch domains of human KLHL2 and KLHL3 are 86% sequence identical. Impaired KLHL3-mediated ubiquitination of WNK4 has been shown to cause hypertension [29].
MDM2_SWIB	5	F[^P]{3}W[^P]{2,3}[VIL]	MDM2 E3 ligase recognizes this motif on the transactivating domain of the p53 family proteins (p53, p63 and p73). This motif region forms an amphipathic α-helix upon binding to the hydrophobic cleft of the MDM2 SWIB domain [30]. Prolines are excluded from the non-conserved positions since they would otherwise disrupt the helical conformation of the degron. The degron is also present in the cell fate determinant protein NUMB and its paralog NUMBL, whose interaction with MDM2 regulated p53 levels by disrupting the p53-MDM2 interaction [31].
Nend_Nbox_1	1	^M{0,1}[FYLIW][^P]	N-end rule pathway degron contain bulky hydrophobic (type II destabilizing residues) at the N-terminus of substrates. Motif is recognized by the N-box domains of N-recognins [32,33].
Nend_UBRbox_1	2	^M{0,1}[RK][^P].	N-end rule pathway degron containing positively charged residues (primary type I destabilizing residues) at the N-terminal, these are specifically recognized by the UBR-box domains of N-recognins. Substrates are often generated by the internal cleavage of a precursor protein [32,33].

Nend_UBRbox_2	1	^M{0,1}([ED]).	This N-degron type has a characteristic Asp/Glu (secondary type I destabilizing residue) at the N-terminal position, that undergoes arginylation (converts the secondary destabilizing residue to primary type I destabilizing residues) to be recognized by UBR-box domains of N-recognins. Like the previous degron, this category can also be generated by internal cleavage of protein precursors [32,33].
Nend_UBRbox_3	1	^M{0,1}([NQ]).	This degron class contains an N-terminal Asn/Gln (tertiary type I destabilizing residue), undergoes deamidation (converts tertiary destabilizing residues to secondary) followed by arginylation (secondary to primary destabilizing residue) to be recognized by UBR-box domains of N-recognins. Once again, this class of degrons can also be generated by internal cleavage of a protein [32,33].
Nend_UBRbox_4	8	^M{0,1}(C).	N-degron (N-terminal) motif on substrates that are recognized by conserved UBRbox domains of specific N-recognins (E3 ligase). The destabilizing N-terminal Cys can be obtained as a result of either N-terminal Met excision or by internal protein cleavage. Proteins with this Cys motif are then oxidized (O_2 and NO-dependent), arginylated and recognized by the UBRbox of N-recognins. This degron occurs in mammalian cells (since NO synthases are required for degron formation) [32,33].
ODPH_VHL_1	8	[IL]A(**P**).{6,8}[FLIVM].[FLIVM]	Hydroxyproline-based degron recognition (under normal oxygen tension) by von Hippel-Lindau tumor suppressor (VHL) protein that is a substrate-recognition component of multi-subunit E3 ligases [34]. Responsible for oxygen-dependent gene expression regulation. Important pathway in the vascularization of solid tumors.
SCF_COI1_1	6	..[RK][RK].SL..F[FLM].[RK]R[HRK].[RK].	Plant-specific degron, plant hormone (eg, auxin, jasmonate) dependent binding to Fbox substrate binding domains of SCF msE3s. COI1 functions as the substrate-recognition subunit of SCF complexes, and mediates binding to jasmonate-dependent degrons found in JAZ family transcriptional repressors. COI1 has a jasmonate-binding site in addition to the degron binding region on its leucine-rich repeat (LRR) domain. The hormone molecule stabilizes the substrate-E3 interface by increasing binding affinity [35].
SCF_FBW7_1	7	[LIVMP].{0,2}(**T**)P..([**ST**])	Phosphodegron motif class, recognized by the WD40 domains of Fbox proteins. Degron found in cell cycle regulatory proteins, contains two phosphorylatable residues that are required for the binding. Mutated phosphodegrons result in enhanced oncogenic function. Widely used in cell cycle regulation [36,37].
SCF_FBW7_2	2	[LIVMP].{0,2}(**T**)P..E	A variant of the above motif where a Glu residue replaces the second phosphosite.
SCF_SKP2-CKS1_1	3	..[DE].(**T**)P.K	Another phosphodegron (singly phosphorylated), here the substrate recognition function is performed by Skp2 (another Fbox protein). Degron recognition occurs via the Leucine Rich Repeats

			(LRR) of Skp2. An additional factor (Cks1) is required to be bound to Skp2 (as a 'pre-assembled' unit) for degron recognition (forming a ternary interface with the substrate degron peptide) [38].
SCF_TIR1_1	7	.[VLIA][VLI]GWPP[VLI]...R.	Auxin-dependent (plant) degron motif found in Aux/IAA transcriptional repressor proteins, bound by the TIR1 F-box proteins (components of SCF msE3s). The substrate degron binds to the composite auxin-TIR1 LRR region [39,40].
SCF-TRCP1	18	D(**S**)G.{2,3}([**ST**])	(Di)phosphodegron recognized by the multi-subunit E3 ligase SCF complex with β-TrCP functioning as the substrate-binding domain. The WD40 domain of β-TrCP binds to the degron using its two phosphate-binding sites. Substrates bearing this motif are important regulators of cell state [41].
SIAH	8	.P.A.V.P[^P]	Degron motif conferring high-affinity binding, it is found in many SIAH E3 ligase substrates that are targeted for proteasomal degradation [42].
SPOP	13	[AVP].[ST][ST][ST]	SPOP functions as a substrate recognition subunit of Cullin3-based, multi-subunit E3 ligase complex by recognizing S/T-rich motifs in its substrates. Unlike many other SCF substrate degrons, phosphorylation of this degron blocks binding to SPOP. A number of SPOP substrates harbor multiple SPOP-binding degrons and the use of multivalent (cooperative) interactions has been suggested for substrate recognition via this degron [43-46].

a The two CBL ligase motifs (PTK and MET) were obtained from Ng et al. [14]. Motif patterns for 5 N-end rule degrons were present in the ELM database [47]; however, 4 of the 5 degron types (Nend_Nbox_1, Nend_UBRbox_1, 2 and 3) had zero instances in ELM. Lists of experimentally validated substrates containing these motifs were therefore obtained from Varshavsky (2011) [32]. The remaining degron categories (and the corresponding target substrates) have been compiled in the ELM database under the "DEG" motif category. Only instances that are experimentally validated to be true positives were included. Details about the motif instances are provided in Supplementary Table 2.

b The motif pattern uses the following nomenclature: "." specifies any amino acid type, "[X]" specifies the allowed amino acid type(s) at that position, "^X" at the beginning of the pattern specifies that the sequence starts with amino acid type X, "[^X]" means that this position can have any amino acid other than type X, numbers specified as the following "X{x,y}", where x and y specify the minimum and maximum number of 'X' amino acid type required at that position. "$" sign implies the carboxy-terminal of the protein chain.

c Conserved residue positions within the primary degron that are known to be post-translationally modified (eg, phosphorylation, proline hydroxylation) are shown in boldface (PTM data from UniProt [48]).

d A more exhaustive description and list of literature references for each of these degrons (except the two CBL E3 ligase motifs) can be obtained from the ELM website (http://elm.eu.org) by searching for "DEG" motifs. The ABBA motif of the APC/C is currently under curation status in the ELM database and therefore accessible from the ELM candidates page (http://elm.eu.org/elms/candidates.html).

Supplementary Table 2. Details of experimentally validated substrates of specific E3 ligases that are targeted using specific linear motifs (primary degrons).

Degron	UniProt ID, substrate name, *organism*	Motif position		Motif sequence
		Start	End	
APC/C (DBOX)	Q9IA80 Securin *X. laevis*	54	62	SRKALGNVN
	P53350 PLK1 kinase *H. sapiens*	336	344	NRKPLTVLN
	Q02363 DNA-binding protein inhibitor ID-2 *H. sapiens*	99	107	SRTPLTTLN
	P07818 G2/mitotic-specific cyclin-B *A. punctulata*	41	49	QRAALGNIS
	O93355 Geminin L *X. laevis*	32	40	PRRTLKVIQ
	P18606 Cyclin-A1 *X. laevis*	40	48	QRTVLGVIG
	Q13309 S-phase kinase-associated protein 2 (Skp2) *H. sapiens*	2	10	HRKHLQEIP
	P14635 G2/mitotic-specific cyclin-B1 *H. sapiens*	41	49	PRTALGDIG
APC/C (KEN)	Q12834 CDC20 *H. sapiens*	96	100	SKENQ
	P24869 G2/mitotic-specific cyclin-2 *S. cerevisiae*	99	103	DKENQ
	Q99741 Cell division control protein 6 homolog *H. sapiens*	80	84	KKENG
	O43683 Mitotic checkpoint serine/threonine-protein kinase	534	538	NKENY
	BUB1 *H. sapiens*	624	628	DKENV
	O95997 Securin *H. sapiens*	8	12	DKENG
	P27895 Kinesin-like protein CIN8 *S. cerevisiae*	931	935	NKENA
	Q96FF9 Sororin *H. sapiens*	87	91	EKENE
	Q8WWK9 Cytoskeleton-associated protein 2 *H. sapiens*	80	84	DKENM
	P30307 M-phase inducer phosphatase 3 *H. sapiens*	150	154	NKEND
	Q96GD4 Aurora kinase B *H. sapiens*	3	7	QKENS
	P34244 Probable serine/threonine-protein kinase HSL1 *S. cerevisiae*	774	778	NKENE
	O60566 Mitotic checkpoint serine/threonine-protein kinase	25	29	SKENV
	BUB1 beta *H. sapiens*	303	307	AKENE
	P03116 Replication protein E1 Bovine papillomavirus type 1	27	31	DKENE
APC/C (ABBA)[a]	P20248 Cyclin-A2 *H. sapiens*	99	107	FTIHVDEAE
APCC_TPR_1	P50082 Meiosis-specific APC/C activator protein AMA1 *S.*	591	593	RIR

	cerevisiae			
	Q9UM13 Anaphase-promoting complex subunit 10 *H. sapiens*	183	185	SIR
	Q3E906 Cell division cycle 20.5 *A. thaliana*	447	449	HIR
	Q12834 Cell division cycle protein 20 homolog *H. sapiens*	497	499	GIR
	Q9SZA4 Cell division cycle 20.1 *A. thaliana*	455	457	RIR
	Q9S7I8 Cell division cycle 20.2 *A. thaliana*	445	447	RIR
	P26309 APC/C activator protein CDC20 *S. cerevisiae*	608	610	LIR
	P53197 APC/C activator protein CDH1 *S. cerevisiae*	564	566	QIR
	Q960N3 cort *D. melanogaster*	481	483	GIR
	Q8LPL5 FIZZY-RELATED 3 *A. thaliana*	479	481	QIR
	Q8L3Z8 FIZZY-RELATED 2 *A. thaliana*	481	483	TIR
	Q9UM11 Fizzy-related protein homolog *H. sapiens*	494	496	RIR
	Q9M2R1 Protein GIGAS CELL1 *A. thaliana*	241	243	TMR
	Q8NI77 Kinesin-like protein KIF18A *H. sapiens*	896	898	NLR
	P51955 Serine/threonine-protein kinase Nek2 *H. sapiens*	443	445	GMR
	Q9M7I2 WD-repeat cell cycle regulatory protein *M. truncatula*	473	475	TIR
CBL (PTK)	P43403 Tyrosine-protein kinase ZAP-70 *H. sapiens*	290	296	DGYTPEP
	O43609 Protein sprouty homolog 1 *H. sapiens*	51	57	NEYTEGP
	O43597 Protein sprouty homolog 2 *H. sapiens*	53	59	NEYTEGP
CBL (MET)	P08581 Hepatocyte growth factor receptor *H. sapiens*	1002	1004	DYR
	P51805 Plexin-A3 *H. sapiens*	1298	1300	DYR
	Q9YGM7 Hepatocyte growth factor receptor *T. rubripes*	1010	1012	DYR
	Q9YGN0 Plasminogen related growth factor receptor 3 *T. rubripes*	997	999	DYR
	Q9YGM5 Plasminogen related growth factor receptor 2 *T. rubripes*	1000	1002	DYR
COP1	O24646 Transcription factor HY5 *A. thaliana*	38	45	EEIRRVPE
	P17535 Transcription factor jun-D *H. sapiens*	241	248	DEPQTVPD
	P05412 Transcription factor AP-1 *H. sapiens*	227	234	EEPQTVPE
	Q9SID1 Salt tolerance-like protein *A. thaliana*	229	236	EEHFLVPD

	P41161 ETS translocation variant 5 *H. sapiens*	66	72	DEQFVPD
	P43268 ETS translocation variant 4 *H. sapiens*	74	80	DEQFVPD
	P50549 ETS translocation variant 1 *H. sapiens*	67	73	DEQFVPD
CRL4_CDT2_1	P38936 Cyclin-dependent kinase inhibitor 1 *H. sapiens*	145	157	TSMTDFYHSKRRL
	P49918 Cyclin-dependent kinase inhibitor 1C *H. sapiens*	270	282	PLISDFFAKRKRS
	Q9H211 DNA replication factor Cdt1 *H. sapiens*	4	15	RRVTDFFARRRP
	Q9I9A7 DNA replication factor Cdt1 *X. laevis*	4	16	MRVTDFFSQSKRG
	Q9NQR1 N-lysine methyltransferase SETD8 *H. sapiens*	220	231	RKLTDFYPVRRS
	Q91603 Xicl protein *X. laevis*	172	184	TPITDYFPKRKKI
CRL4_CDT2_2	Q27368 Transcription factor E2f *D. melanogaster*	151	163	NDITNYYKVKRRP
Kelch_KEAP1_1	Q16236 Nuclear factor erythroid 2-related factor 2 *H. sapiens*	77	82	DEETGE
	Q96HS1 Serine/threonine-protein phosphatase PGAM5 *H. sapiens*	77	82	NVESGE
	O14920 Inhibitor of nuclear factor kappa-B kinase subunit beta *H. sapiens*	34	39	NQETGE
	Q64337 Sequestosome-1 *M. musculus*	349	354	DPSTGE
	P26350 Prothymosin alpha *M. musculus*	41	46	NEENGE
	Q12830 Nucleosome-remodeling factor subunit BPTF *H. sapiens*	729	734	DPENGE
	Q13402 Unconventional myosin-VIIa *H. sapiens*	1636	1641	DHDTGE
	Q86YC2 Partner and localizer of BRCA2 *H. sapiens*	89	94	DEETGE
	Q5JTC6 APC membrane recruitment protein 1 *H. sapiens*	286	291	SPETGE
	Q13501 Sequestosome-1 *H. sapiens*	347	352	DPSTGE
	P20482 Segmentation protein cap'n'collar *D. melanogaster*	458	463	DNETGE
	Q14494 Nuclear factor erythroid 2-related factor 1 *H. sapiens*	231	236	DGETGE
	Q60795 Nuclear factor erythroid 2-related factor 2 *M. musculus*	77	82	DEETGE
Kelch_KEAP1_2	Q60795 Nuclear factor erythroid 2-related factor 2 *M. musculus*	26	32	QDIDLGV
Kelch_actinfilin	P42260 Glutamate receptor ionotropic, kainate 2 *R. norvegicus*	881	885	APVIV
Kelch_KLHL3	Q9H4A3 Serine/threonine-protein kinase WNK1 *H. sapiens*	628	637	EPEEPEADQH
	Q9Y3S1 Serine/threonine-protein kinase WNK2 *H. sapiens*	588	597	EPEEPEADQH
	Q9BYP7 Serine/threonine-protein kinase WNK3 *H. sapiens*	537	546	ECEETEVDQH
	Q96J92 Serine/threonine-protein kinase WNK4 *H. sapiens*	557	566	EPEEPEADQH

MDM2_SWIB	Q9H3D4 Tumor protein 63 *H. sapiens*<br>O15350 Tumor protein p73 *H. sapiens*<br>P04637 Cellular tumor antigen p53 *H. sapiens*<br>P49757 Protein numb homolog *H. sapiens*<br>Q9Y6R0 Numb-like protein *H. sapiens*	55<br>15<br>19<br>616<br>577	62<br>22<br>26<br>623<br>584	FQHIWDFL<br>FEHLWSSL<br>FSDLWKLL<br>FEAQWAAL<br>FEAQWAAL
Nend_Nbox_1	P03367 HIV integrase (cleavage product of Gag-Pol polyprotein) *Human immunodeficiency virus type 1*	1160	1161	FL
Nend_UBRbox_1	Q12158 Sister chromatid cohesion protein 1 *S. cerevisiae*<br>P13128 Listeriolysin O *L. monocytogenes*	181<br>25	183<br>27	RFS<br>KDA
Nend_UBRbox_2	O60216 Double-strand-break repair protein rad21 homolog *H. sapiens*	173	174	EG
Nend_UBRbox_3	Q24306 Apoptosis 1 inhibitor *D. melanogaster*	21	22	NN
Nend_UBRbox_4	O22259 Ethylene-responsive transcription factor ERF071 *A. thaliana*<br>Q9SSA8 Ethylene-responsive transcription factor RAP2-12 *A. thaliana*<br>Q9LUM4 Ethylene-responsive transcription factor RAP2-2 *A. thaliana*<br>P42736 Ethylene-responsive transcription factor RAP2-3 *A. thaliana*<br>P97428 Regulator of G-protein signaling 16 *M. musculus*<br>O08899 Regulator of G-protein signaling 4 *M. musculus*<br>O08850 Regulator of G-protein signaling 5 *M. musculus*<br>F4IDA7 Ethylene-responsive transcription factor ERF073 *A. thaliana*	1<br>1<br>1<br>1<br>1<br>1<br>1<br>1	3<br>3<br>3<br>3<br>3<br>3<br>3<br>3	MCG<br>MCG<br>MCG<br>MCG<br>MCR<br>MCK<br>MCK<br>MCG
ODPH_VHL_1	Q99814 Endothelial PAS domain-containing protein 1 *H. sapiens*<br><br><br>Q16665 Hypoxia-inducible factor 1-alpha *H. sapiens*	529<br>403<br>574<br>400<br>562	542<br>416<br>585<br>413<br>574	LAPYIPMDGEDFQL<br>LAPTPGDAIISLDF<br>LAPVAPHSPFLL<br>LAPAAGDTIISLDF<br>LAPYIPMDDDFQL

	G5EGD2 Hypoxia-inducible factor 1 *C. elegans*<br>Q9Y2N7 Hypoxia-inducible factor 3-alpha *H. sapiens*<br>Q24167 sima *D. melanogaster*	619<br>490<br>1173	631<br>502<br>1184	LAPFVDTYDMMQM<br>LAPYISMDDDFQL<br>IAPVNTKATIRL
SCF_COI1_1	Q9LVI4 Protein TIFY 6B *A. thaliana*<br>Q9LMA8 Protein TIFY 10A *A. thaliana*<br>Q8W4J8 Protein TIFY 7 *A. thaliana*<br>Q9S7M2 Protein TIFY 10B *A. thaliana*<br>A7XXZ0 Jasmonate ZIM-domain protein 1 *S. lycopersicum*<br>B2XVS2 Jasmonate ZIM-domain protein 3 *S. lycopersicum*	303<br>203<br>221<br>205<br>199<br>251	320<br>220<br>238<br>222<br>216<br>268	LARKASLARFLEKRKERV<br>IARRASLHRFLEKRKDRV<br>QARKASLARFLEKRKERL<br>IARRASLHRFLEKRKDRI<br>IARRNSLTRFLEKRKDRV<br>QARKASLARFLEKRKERV
SCF_FBW7_1	P24864 G1/S-specific cyclin-E1 *H. sapiens*<br>P24864-3 G1/S-specific cyclin-E1 isoform-3 *H. sapiens*<br>P05412 Transcription factor AP-1 *H. sapiens*<br>P01106 Myc proto-oncogene protein *H. sapiens*<br>P36956 Sterol regulatory element-binding protein 1 *H. sapiens*<br>P13051 Uracil-DNA glycosylase *H. sapiens*<br>P38634 Protein SIC1 *S. cerevisiae*	393<br>379<br>236<br>55<br>425<br>58<br>43	399<br>384<br>243<br>62<br>430<br>64<br>49	LLTPPQS<br>LTPPQS<br>PGETPPLS<br>LLPTPPLS<br>LTPPPS<br>PGTPPSS<br>PVTPSTT
SCF_FBW7_2	P03070 Large T antigen *Simian virus 40*<br>P46531 Neurogenic locus notch homolog protein 1 *H. sapiens*	699<br>2508	705<br>2515	PPTPPPE<br>PFLTPSPE
SCF_SKP2-<br>CKS1_1	P46527 Cyclin-dependent kinase inhibitor 1B *H. sapiens*<br>P49918 Cyclin-dependent kinase inhibitor 1C *H. sapiens*<br>P49919 Cyclin-dependent kinase inhibitor 1C *M. musculus*	183<br>306<br>338	190<br>313<br>345	SVEQTPKK<br>SVEQTPRK<br>AVEQTPRK
SCF_TIR1_1	Q38825 Auxin-responsive protein IAA7 *A. thaliana*<br>P93830 Auxin-responsive protein IAA17 *A. thaliana*<br>P49677 Auxin-responsive protein IAA1 *A. thaliana*<br>Q9XFM0 Auxin-responsive protein IAA28 *A. thaliana*<br>Q38830 Auxin-responsive protein IAA12 *A. thaliana*<br>Q38822 Auxin-responsive protein IAA3 *A. thaliana*<br>P49680 Auxin-induced protein IAA6 *P. sativum*	82<br>82<br>55<br>48<br>69<br>64<br>55	94<br>94<br>67<br>60<br>81<br>76<br>67	QVVGWPPVRNYRK<br>QVVGWPPVRSYRK<br>QIVGWPPVRSNRK<br>PVVGWPPVRSSRR<br>QVVGWPPIGLHRM<br>QIVGWPPVRSYRK<br>QVVGWPPVCSYRK
SCF_TRCP1	Q15653 NF-kappa-B inhibitor beta *H. sapiens*<br>O00221 NF-kappa-B inhibitor epsilon *H. sapiens*	18<br>156	23<br>161	DSGLGS<br>DSGIES

	P35222 Catenin beta-1 *H. sapiens*	32	37	DSGIHS
	P19838 Nuclear factor NF-kappa-B p105 subunit *H. sapiens*	926	932	DSGVETS
	P25963 NF-kappa-B inhibitor alpha *H. sapiens*	31	36	DSGLDS
	Q9HAW4 Claspin *H. sapiens*	29	34	DSGQGS
	P18848 Cyclic AMP-dependent transcription factor ATF-4 *H. sapiens*	218	224	DSGICMS
	Q12959 Disks large homolog 1 *H. sapiens*	597	602	DSGLPS
	Q9UKT4 F-box only protein 5 *H. sapiens*	144	149	DSGYSS
	O95863 Zinc finger protein SNAI1 *H. sapiens*	95	100	DSGKGS
	Q6PGQ7 Protein aurora borealis *H. sapiens*	496	501	DSGYNT
	P16471 Prolactin receptor *H. sapiens*	348	353	DSGRGS
	P05923 Protein Vpu *Human immunodeficiency virus type 1*	51	56	DSGNES
	P03230 Latent membrane protein 1 *Epstein-Barr virus*	210	215	DSGHES
	Q53EL6 Programmed cell death protein 4 *H. sapiens*	70	76	DSGRGDS
	P98174 FYVE, RhoGEF and PH domain-containing protein 1 *H. sapiens*	282	287	DSGIDS
	Q5JSP0 FYVE, RhoGEF and PH domain-containing protein 3 *H. sapiens*	75	80	DSGIDS
	P17181 Interferon alpha/beta receptor 1 *H. sapiens*	534	539	DSGNYS
SIAH	Q16633 POU domain class 2-associating factor 1 *H. sapiens*	46	54	APTAVVLPH
	P43146 Netrin receptor DCC *H. sapiens*	1331	1339	IPTACVRPT
	Q9UHB7 AF4/FMR2 family member 4 *H. sapiens*	252	260	KPTAYVRPM
	O75553 Disabled homolog 1 *H. sapiens*	360	368	PPVAQVMPG
	Q9HB71 Calcyclin-binding protein *H. sapiens*	59	67	KPAAVVAPI
	Q13118 Krueppel-like factor 10 *H. sapiens*	200	208	IPCAAVSPN
	Q7Z6J0 E3 ubiquitin-protein ligase SH3RF1 *H. sapiens*	600	608	RPTAAVTPI
	Q86TG7 Retrotransposon-derived protein PEG10 *H. sapiens*	253	261	PPRALVLPH
SPOP[b]	Q9VHV8 Puckered, isoform A *D. melanogaster*	98<br>289<br>381	102<br>293<br>385	VTSTT<br>PSSSS<br>PSSTS

	O75367 Core histone macro-H2A.1 *H. sapiens*<br>P19538 Transcriptional activator cubitus interruptus *D. melanogaster*<br>Q9UER7 Death domain-associated protein 6 *H. sapiens*<br><br>P10275 Androgen receptor *H. sapiens*<br>P03372 Estrogen receptor *H. sapiens*<br>Q0VGT2 Zinc finger protein GLI2 *M. musculus*<br>P10071 Transcriptional activator GLI3 *H. sapiens*	171<br>371<br>1362<br>608<br>680<br>645<br>571<br>1177<br>161<br>422	175<br>375<br>1366<br>612<br>684<br>649<br>575<br>1181<br>165<br>426	ADSTT<br>PSSTS<br>VSSST<br>VSSTS<br>ADSST<br>ASSTT<br>AGSTS<br>VQSSS<br>ALSSS<br>AVSST

a The ABBA motif has dual inhibitory and degron roles for the APC/C [11]. Only the degradation substrate (Cyclin A) is listed here (as well as in Table 1 and Supplementary Table 1), since we are focusing on the degron role. However, the motif pattern is derived using all characterized ABBA instances (human cyclin A2, BubR1, Bub1 and yeast Acm1).

b The SPOP binding motif is enriched in Ser and Thr residues and many SPOP substrates contain multiple Ser/Thr-rich regions that act cooperatively to bind SPOP. Only those motifs are included here that have: 1) been experimentally demonstrated (mostly by mutagenesis experiments) to be required for SPOP binding, and 2) match the current ELM consensus motif pattern. For most of the substrates, the ones included here correspond to the most crucial motifs for SPOP binding and degradation.

Supplementary Table 3. PDB structures of unbound (free) substrates with an experimentally validated primary degron. These PDB structures were selected because the constructs used for the structure determination experiment contained the primary degron sequence. However, only the structures of the Kelch_KEAP1_1 substrate I-kappa-B-kinase beta (UniProt ID: O14920; PDB IDs: 4E3C, 4KIK) have visible electron density for the degron region.

Primary degron	UniProt ID	PDB code
Apcc_tpr_1	Q12834	4GGA
KEN	Q12834	4GGA
Dbox	P53350	2OWB
Dbox	P53350	2OU7
Dbox	P53350	4A4O
Dbox	P53350	3FC2
Dbox	P53350	3KB7
Dbox	P53350	2YAC
Dbox	P53350	3THB
Dbox	P53350	4A4L
Kelch_KEAP1_1	O14920	4E3C, 4KIK
Nend_ubrbox_3	Q24306	3SIQ
PTK	P43403	2OZO
PTK	P43403	4K2R
SIAH	Q9HB71	1X5M
SPOP	O75367	1ZR5
Kelch_actinfilin	P42260	4UQQ

Supplementary Table 4. Non-redundant list of PDB structures for substrate primary degrons in complex with E3 ligases (or with substrate recognition adaptor subunits in case of multi-subunit E3 complexes).

PDB code (ChainIDs Substrate degron: E3 component)	Degron containing substrate (UniProt ID)	Substrate sequence present in PDB file (sequence of known degron shown in **red**)	Degron type	E3 ligase/substrate adaptor subunit (UniProt ID)
4GGD_D:B 4GGD_C:A	BUB1B_HUMAN (O60566)	L**SKEN**VQ	APC/C (KEN)	CDC20_HUMAN (Q12834)
1YCR_B:A	P53_HUMAN (P04637)	ET**FSDLWKLL**PEN	MDM2	MDM2_HUMAN (Q00987)
2MPS_B:A	P73_HUMAN (O15350)	DGGTT**FEHLWSSL**EPD	MDM2	MDM2_HUMAN (Q00987)
1LM8_H:V	HIF1A_HUMAN (Q16665)	M**LAPYIPMDDDFQL**R	ODPH_VHL_1	VHL_HUMAN (P40337)
2FLU_P:X	NF2L2_HUMAN (Q16236)	AFFAQLQL**DEETGE**FL	Kelch_KEAP1_1	KEAP1_HUMAN (Q14145)
1X2R_B:A	NF2L2_MOUSE (Q60795)	L**DEETGE**FL	Kelch_KEAP1_1	KEAP1_MOUSE (Q9Z2X8)
2Z32_B:A	PTMA_MOUSE (P26350)	Q**NEENGE**QE	Kelch_KEAP1_1	KEAP1_MOUSE (Q9Z2X8)
3ADE_B:A	SQSTM_MOUSE (Q64337)	V**DPSTGE**L	Kelch_KEAP1_1	KEAP1_MOUSE (Q9Z2X8)
3WN7_B:A	NF2L2_MOUSE (Q60795)	MDLIDILWR**QDIDLGV**SREVFDF S	Kelch_KEAP1_2	KEAP1_MOUSE (Q9Z2X8)
4CHB_C:A	WNK4_HUMAN (Q96J92)	**EPEEPEADQH**	Kelch_KLHL3	KLHL2_HUMAN (O95198)
3OGL_Q:B	TI10A_ARATH (Q9LMA8)	ELP**IARRASLHRFLEKRK**	SCF_COI1_1	COI1_ARATH (O04197)
2OVQ_C:B	CCNE1_HUMAN (P24864)	LPSG**LLTPPQS**G	SCF_FBW7_1	FBXW7_HUMAN (Q969H0)
1MV0_A:B	MYC_HUMAN (P01106)	**LLPTPPLS**PSRRSG	SCF_FBW7_1	BIN1_HUMAN (O00499)
2AST_D:C	CDN1B_HUMAN (P46527)	AG**SVEQTPKK**	SCF_SKP2-CKS1_1	CKS1_HUMAN (P61024)
2P1Q_C:B	IAA7_ARATH (Q38825)	**QVVGWPPVRNYRK**	SCF_TIR1_1	TIR1_ARATH (Q570C0)
1P22_C:A	CTNB1_HUMAN (P35222)	YL**DSGIHS**GAT	SCF_TRCP1	FBW1A_HUMAN (Q9Y297)
2A25_B:A	CYBP_HUMAN (Q9HB71)	**KPAAVVAPI**	SIAH	SIAH1_HUMAN (Q8IUQ4)
2CBL_B:A	ZAP70_HUMAN (P43403)	S**DGYTPEP**A	CBL (PTK)	CBL_HUMAN (P22681)
3OB1_A:B	SPY2_HUMAN (O43597)	RNT**NEYTEGP**	CBL (PTK)	CBL_HUMAN (P22681)
3BUX_A:B	MET_HUMAN (P08581)	NESV**DYR**A	CBL (MET)	CBL_HUMAN (P22681)

3HQM_C:A	CI_DROME (P19538)	PD**VSSST**	SPOP	SPOP_HUMAN (O43791)
3IVB_M:A	H2AY_HUMAN (O75367)	AS**ADSTT**EGTP	SPOP	SPOP_HUMAN (O43791)
3HQL_C:A	Q9VHV8_DROME (Q9VHV8)	CDE**VTSTT**	SPOP	SPOP_HUMAN (O43791)

For 4GGD.pdb, there are two degron-E3 complexes in the asymmetric unit and the degron peptide is found in different structural conformations (a 3-10 helix and a turn conformation) in the two complexes. Therefore both are indicated in this table and both degron conformations have been used for making the overall statistics shown in Supplementary Figure 3.

Supplementary Table 5. Non-redundant PDB structures for secondary degrons (Deg lysines).

Protein (UniProt ID)	PDB code	Residue number of Deg lysine(s) visible in PDB structure
Cellular tumor antigen p53 (P04637)	1OLG	319, 320, 321
Cellular tumor antigen p53 (P04637)	2PCX	101, 120, 132, 139, 164, 291, 292
Cyclin-dependent kinase 4 inhibitor D (P55273)	1BD8	62
Nuclear factor NF-kappa-B p100 subunit (Q00653)	2D96	855 in UniProt sequence (res. 97 in PDB file)
E3 ubiquitin-protein ligase Mdm2 (Q00987)	2HDP	446
Induced myeloid leukemia cell differentiation protein Mcl-1 (Q07820)	3WIX	194, 197
ELAV-like protein 1 (Q15717)	4EGL	182
Ubiquitin carboxyl-terminal hydrolase 7 (Q93009)	2YLM	869
Suppressor of fused homolog (Q9UMX1)	4KM8	257

References

1 Vaquerizas, J. M., Kummerfeld, S. K., Teichmann, S. A. & Luscombe, N. M. A census of human transcription factors: function, expression and evolution. *Nat Rev Genet* **10**, 252-263 (2009).

2 Consortium, E. P. *et al.* An integrated encyclopedia of DNA elements in the human genome. *Nature* **489**, 57-74 (2012).

3 Manning, G., Whyte, D. B., Martinez, R., Hunter, T. & Sudarsanam, S. The protein kinase complement of the human genome. *Science* **298**, 1912-1934 (2002).

4 Choudhary, C. & Mann, M. Decoding signalling networks by mass spectrometry-based proteomics. *Nat Rev Mol Cell Biol* **11**, 427-439 (2010).

5 Tompa, P., Davey, N. E., Gibson, T. J. & Babu, M. M. A Million Peptide Motifs for the Molecular Biologist. *Mol Cell* **55**, 161-169 (2014).

6 Davey, N. E., Haslam, N. J., Shields, D. C. & Edwards, R. J. SLiMSearch 2.0: biological context for short linear motifs in proteins. *Nucleic Acids Res* **39**, W56-60 (2011).

7 Cilia, E., Pancsa, R., Tompa, P., Lenaerts, T. & Vranken, W. F. The DynaMine webserver: predicting protein dynamics from sequence. *Nucleic Acids Res* **42**, W264-270 (2014).

8 Samanta, U., Bahadur, R. P. & Chakrabarti, P. Quantifying the accessible surface area of protein residues in their local environment. *Protein Eng* **15**, 659-667 (2002).

9 Peters, J. M. The anaphase promoting complex/cyclosome: a machine designed to destroy. *Nat Rev Mol Cell Biol* **7**, 644-656 (2006).

10 Castro, A., Bernis, C., Vigneron, S., Labbe, J. C. & Lorca, T. The anaphase-promoting complex: a key factor in the regulation of cell cycle. *Oncogene* **24**, 314-325 (2005).

11 Di Fiore, B. *et al.* The ABBA motif binds APC/C activators and is shared by APC/C substrates and regulators. *Dev Cell* **32**, 358-372 (2015).

12 Sedgwick, G. G. *et al.* Mechanisms controlling the temporal degradation of Nek2A and Kif18A by the APC/C-Cdc20 complex. *EMBO J* **32**, 303-314 (2013).

13 Matyskiela, M. E. & Morgan, D. O. Analysis of activator-binding sites on the APC/C supports a cooperative substrate-binding mechanism. *Mol Cell* **34**, 68-80 (2009).

14 Ng, C. *et al.* Structural basis for a novel intrapeptidyl H-bond and reverse binding of c-Cbl-TKB domain substrates. *EMBO J* **27**, 804-816 (2008).
15 Schmidt, M. H. & Dikic, I. The Cbl interactome and its functions. *Nat Rev Mol Cell Biol* **6**, 907-918 (2005).
16 Peschard, P., Ishiyama, N., Lin, T., Lipkowitz, S. & Park, M. A conserved DpYR motif in the juxtamembrane domain of the Met receptor family forms an atypical c-Cbl/Cbl-b tyrosine kinase binding domain binding site required for suppression of oncogenic activation. *J Biol Chem* **279**, 29565-29571 (2004).
17 Peschard, P. *et al.* Mutation of the c-Cbl TKB domain binding site on the Met receptor tyrosine kinase converts it into a transforming protein. *Mol Cell* **8**, 995-1004 (2001).
18 Dornan, D. *et al.* The ubiquitin ligase COP1 is a critical negative regulator of p53. *Nature* **429**, 86-92 (2004).
19 Serino, G. & Deng, X. W. The COP9 signalosome: regulating plant development through the control of proteolysis. *Annu Rev Plant Biol* **54**, 165-182 (2003).
20 Ang, L. H. *et al.* Molecular interaction between COP1 and HY5 defines a regulatory switch for light control of Arabidopsis development. *Mol Cell* **1**, 213-222 (1998).
21 Havens, C. G. & Walter, J. C. Docking of a specialized PIP Box onto chromatin-bound PCNA creates a degron for the ubiquitin ligase CRL4Cdt2. *Mol Cell* **35**, 93-104 (2009).
22 Lo, S. C., Li, X., Henzl, M. T., Beamer, L. J. & Hannink, M. Structure of the Keap1:Nrf2 interface provides mechanistic insight into Nrf2 signaling. *EMBO J* **25**, 3605-3617 (2006).
23 Lo, S. C. & Hannink, M. PGAM5, a Bcl-XL-interacting protein, is a novel substrate for the redox-regulated Keap1-dependent ubiquitin ligase complex. *J Biol Chem* **281**, 37893-37903 (2006).
24 Lee, D. F. *et al.* KEAP1 E3 ligase-mediated downregulation of NF-kappaB signaling by targeting IKKbeta. *Mol Cell* **36**, 131-140 (2009).
25 Padmanabhan, B. *et al.* Structural basis for defects of Keap1 activity provoked by its point mutations in lung cancer. *Mol Cell* **21**, 689-700 (2006).
26 Katoh, Y. *et al.* Evolutionary conserved N-terminal domain of Nrf2 is essential for the Keap1-mediated degradation of the protein by proteasome. *Arch Biochem Biophys* **433**, 342-350 (2005).
27 Salinas, G. D. *et al.* Actinfilin is a Cul3 substrate adaptor, linking GluR6 kainate receptor subunits to the ubiquitin-proteasome pathway. *J Biol Chem* **281**, 40164-40173 (2006).
28 Takahashi, D. *et al.* KLHL2 interacts with and ubiquitinates WNK kinases. *Biochem Biophys Res Commun* **437**, 457-462 (2013).
29 Wakabayashi, M. *et al.* Impaired KLHL3-mediated ubiquitination of WNK4 causes human hypertension. *Cell Rep* **3**, 858-868 (2013).

30 Kussie, P. H. *et al.* Structure of the MDM2 oncoprotein bound to the p53 tumor suppressor transactivation domain. *Science* **274**, 948-953 (1996).
31 Sczaniecka, M. *et al.* MDM2 protein-mediated ubiquitination of numb protein: identification of a second physiological substrate of MDM2 that employs a dual-site docking mechanism. *J Biol Chem* **287**, 14052-14068 (2012).
32 Varshavsky, A. The N-end rule pathway and regulation by proteolysis. *Protein Sci* **20**, 1298-1345 (2011).
33 Tasaki, T., Sriram, S. M., Park, K. S. & Kwon, Y. T. The N-end rule pathway. *Annu Rev Biochem* **81**, 261-289 (2012).
34 Min, J. H. *et al.* Structure of an HIF-1alpha -pVHL complex: hydroxyproline recognition in signaling. *Science* **296**, 1886-1889 (2002).
35 Sheard, L. B. *et al.* Jasmonate perception by inositol-phosphate-potentiated COI1-JAZ co-receptor. *Nature* **468**, 400-405 (2010).
36 Hao, B., Oehlmann, S., Sowa, M. E., Harper, J. W. & Pavletich, N. P. Structure of a Fbw7-Skp1-cyclin E complex: multisite-phosphorylated substrate recognition by SCF ubiquitin ligases. *Mol Cell* **26**, 131-143 (2007).
37 Welcker, M. & Clurman, B. E. FBW7 ubiquitin ligase: a tumour suppressor at the crossroads of cell division, growth and differentiation. *Nat Rev Cancer* **8**, 83-93 (2008).
38 Hao, B. *et al.* Structural basis of the Cks1-dependent recognition of p27(Kip1) by the SCF(Skp2) ubiquitin ligase. *Mol Cell* **20**, 9-19 (2005).
39 Tan, X. *et al.* Mechanism of auxin perception by the TIR1 ubiquitin ligase. *Nature* **446**, 640-645 (2007).
40 Parry, G. & Estelle, M. Auxin receptors: a new role for F-box proteins. *Curr Opin Cell Biol* **18**, 152-156 (2006).
41 Wu, G. *et al.* Structure of a beta-TrCP1-Skp1-beta-catenin complex: destruction motif binding and lysine specificity of the SCF(beta-TrCP1) ubiquitin ligase. *Mol Cell* **11**, 1445-1456 (2003).
42 House, C. M. *et al.* A binding motif for Siah ubiquitin ligase. *Proc Natl Acad Sci U S A* **100**, 3101-3106 (2003).
43 Zhuang, M. *et al.* Structures of SPOP-substrate complexes: insights into molecular architectures of BTB-Cul3 ubiquitin ligases. *Mol Cell* **36**, 39-50 (2009).
44 Zhang, Q. *et al.* Multiple Ser/Thr-rich degrons mediate the degradation of Ci/Gli by the Cul3-HIB/SPOP E3 ubiquitin ligase. *Proc Natl Acad Sci U S A* **106**, 21191-21196 (2009).
45 An, J., Wang, C., Deng, Y., Yu, L. & Huang, H. Destruction of full-length androgen receptor by wild-type SPOP, but not prostate-cancer-associated mutants. *Cell Rep* **6**, 657-669 (2014).
46 Zhang, P. *et al.* Endometrial cancer-associated mutants of SPOP are defective in regulating estrogen receptor-alpha protein turnover. *Cell Death Dis* **6**, e1687 (2015).
47 Dinkel, H. *et al.* The eukaryotic linear motif resource ELM: 10 years and counting. *Nucleic Acids Res* **42**, D259-266 (2014).
48 UniProt, C. Activities at the Universal Protein Resource (UniProt). *Nucleic Acids Res* **42**, D191-198 (2014).
